# Supplementary material for: A Patient-Centered Documentation Skills Curriculum for Preclerkship Medical Students in an Open Notes Era
Source: MedEdPORTAL. 2024 Mar 26;20:11392. doi: 10.15766/mep_2374-8265.11392 (PMC10963659; doi:10.15766/mep_2374-8265.11392)
Supplement: Supplementary file 1 — Checklist of Best Practices.docxRubric.docxFacilitator Guide.docxCourse Planner Implementation Guide.docxAsynchronous Module folderStudent Guide.docxWritten Documentation Guide.docxStudent Session Slides.pptxSample Note.docxModel Note.docxAttitudinal Survey Questions.docxKnowledge Questions.docx [file mep_2374-8265.11392-s001.zip › _Educational Summary Report_11392.pdf]

MedEdPORTAL® The AAMC Journal of Teaching and Learning Resources

Original Publication

Open Access

A Patient-Centered Documentation Skills Curriculum for Preclerkship Medical Students in an Open Notes Era

Kathleen Eng*, Katherine Johnston, MD, Ivo Cerda, MSE, Kushal Kadakia, MSE, MSt, Alison Mosier-Mills, Anita Vanka, MD

*Corresponding author: kathleen_eng@hms.harvard.edu

Abstract

Introduction: New legislation allows patients (with permitted exceptions) to read their clinical notes, leading to both benefits and ethical dilemmas. Medical students need a robust curriculum to learn documentation skills within this challenging context. We aimed to teach note-writing skills through a patient-centered lens with special consideration for the impact on patients and providers. We developed this session for first-year medical students within their foundational clinical skills course to place bias-free language at the forefront of how they learn to construct a medical note. **Methods:** One hundred seventy-three first-year medical and dental students participated in this curriculum. They completed an asynchronous presession module first, followed by a 2-hour synchronous workshop including a didactic, student-led discussion and sample patient note exercise. Students were subsequently responsible throughout the year for constructing patient-centered notes, graded by faculty with a newly developed rubric and checklist of best practices. **Results:** On postworkshop surveys, learners reported increased preparedness in their ability to document in a patient-centered manner (presession $M = 2.2$, midyear $M = 3.9$, $p < .001$), as rated on a 5-point Likert scale (1 = *not prepared at all,* 5 = *very prepared*), and also found this topic valuable to learn early in their training. **Discussion:** This curriculum utilizes a multipart approach to prepare learners to employ clinical notes to communicate with patients and providers, with special attention to how patients and their care partners receive a note. Future directions include expanding the curriculum to higher levels of learning and validating the developed materials.

Keywords
Bias-Free Language, Documentation, Electronic Health Record, Medical Notes, Open Notes, Patient-Centered, Bias, Clinical/Procedural Skills Training

Educational Objectives

By the end of this activity, learners will be able to:

1. Define the 21st Century Cures Act and the research of open notes and patient engagement.
2. Identify language used in discussing patients and documenting patient concerns that could be harmful to patients, create unwanted bias, or adversely affect other health professionals' understanding of patients.
3. Demonstrate a patient-centered approach to documentation and increase self-reported preparedness in using nondiscriminatory, nonjudgmental, and inclusive language in clinical notes.

Citation:
Eng K, Johnston K, Cerda I, Kadakia K, Mosier-Mills A, Vanka A. A patient-centered documentation skills curriculum for preclerkship medical students in an open notes era. *MedEdPORTAL.* 2024;20:11392. https://doi.org/10.15766/mep_2374-8265.11392

Introduction

Since the implementation of the 21st Century Cures Act in April 2021, federal rules mandate that all patients (with several permitted exceptions) be offered rapid, online access to their clinical records, including the notes written by clinicians (open notes).[1] Research demonstrates that patients who read their notes feel more involved in and knowledgeable about their care, are better prepared for visits, and are more likely to follow their clinicians' advice.[2-4] While this change has created significant opportunities to strengthen patient-clinician teamwork and patient engagement in health care, it has also left providers and learners feeling ill-equipped to tackle its novel ethical and practical challenges.[5] Trainees need a dedicated curriculum on learning documentation skills through a patient-centered lens so that they can develop documentation skills that rise to the ethical and practical standards of an open notes era.

In a 2022 cross-sectional study, one in 40 hospital admission notes included stigmatizing language.[6] Words matter, and what

Copyright © 2024 Eng et al. This is an open-access publication distributed under the terms of the Creative Commons Attribution license.

historically has been the norm for medical vernacular (e.g., *patient denies, obesity*) now risks offending the patient as a reader, creating unwanted bias and jeopardizing the physician-patient relationship. Stigmatizing notes can also impact trust with patients from various sexual/gender, cultural, ethnic, or religious minorities, as well as those with substance use disorders.[7,8] From a practical standpoint, since the implementation of open notes, studies show that clinicians report being less candid in documentation, and about a quarter have changed how they write differential diagnoses.[9,10] Providers are challenged to balance communicating clinical data in a manner that averts unintended harm or worry while still maintaining high clinical accuracy and quality.

According to the AAMC guidelines for core entrustable professional activities (EPAs) for entering residency, documentation of a clinical encounter is a core competency for medical students (EPA 5).[11] Therefore, there exists a need to teach medical students documentation skills within the context of this challenging environment. While various curricula are designed to teach documentation skills, most have been developed with an emphasis on specific clinical settings[12,13] or focus on practical skills and efficiency.[6,14-16] Movements to focus on bias and stigmatizing language in medical education are reflected in a few recent curricula published in *MedEdPORTAL*. For example, Stagno, Crapanzano, and Schwartz created a workshop to facilitate learning about patient-centered communication in a mental health setting, developing a mindful language tool kit to provide tips on language best practices.[17] Raney and colleagues developed a workshop to address biased and stigmatizing language using a framework that reinforces antibias and antioppressive skills.[18] However, both curricula are designed for broad audiences and disseminated as elective workshops. There is a need for a robust curriculum that engages every medical student in patient-centered documentation during training. Our curriculum is the first to our knowledge to introduce learners to best-practice documentation skills while, in parallel, challenging them to understand key ethical considerations and cross-examine the impact of open notes from both patient and provider perspectives.

We designed this longitudinal curriculum to be interwoven with the 11-month foundational clinical skills course taught to 173 first-year medical and dental students at our medical school. We specifically targeted first-year students to keep the ethical considerations of open notes and importance of bias-free language at the forefront of how they learn to construct a medical note. The curriculum is structured on Bloom's taxonomy as revised by Anderson and Krathwohl to enforce learning.[19] Students obtain knowledge and test recollection during asynchronous prework, followed by application, analysis, and evaluation of knowledge during the synchronous session. Active learning strategies such as pause procedures with pair brainstorming and case-based learning optimize learner engagement.[20] Finally, learners employ skills throughout the year, writing clinical notes, with our newly developed checklist of best practices and rubric as the cornerstone of evaluation.

Therefore, this curriculum provides first-year students with the fundamental skills to accurately communicate clinical encounters while encouraging a systemic change using patient-centered and nonstigmatizing language in documented clinical encounters. The curriculum is poised to keep up with the demands of a rapidly evolving clinical environment, emboldening future clinicians to communicate thoughtfully with patients and providers.

Methods

Curriculum Design

We designed this educational innovation in collaboration with faculty and a medical student task force at Harvard Medical School (HMS). We conducted a literature review and four focus groups to identify key themes necessary for our curriculum. Focus groups consisted of internal medicine residents, patient advocates, national physician experts in OpenNotes,[2] and medical student educators.

We designed the cornerstones of our curriculum, the checklist of best practices and patient-centered documentation rubric (Appendices A and B), in collaboration with medical educators, OpenNotes faculty and consultants, and patient advocates to address difficult ethical issues and meet the needs of diverse patient populations. We also piloted these materials with a medical student educator group to elicit feedback. We designed the patient-centered documentation rubric as a modification of the IDEA (interpretive summary, differential diagnosis, explanation of reasoning, and alternatives) assessment tool.[21] The modification added a section that included concepts of patient centeredness. It also removed the requirement for assessments and plans, as these aspects were a later focus beyond our preclerkship course. When piloted with course faculty and clinical preceptors, the focus on history of present illness (HPI), medical history, and physical examination seemed appropriate for our foundational skills course and took preceptors 5 minutes to complete. Prior to the course, faculty preceptors had an instructional workshop to review all curricular components and practice use of the rubric (Appendices C and D).

Copyright © 2024 Eng et al. This is an open-access publication distributed under the terms of the Creative Commons Attribution license.

Setting and Participants

We implemented the curriculum for the first-year medical and dental student class enrolled in the HMS Practice of Medicine course, a weekly longitudinal clinical skills course teaching the foundational skills of interview and communication, physical exam, clinical reasoning, oral presentation, and documentation during the preclerkship curriculum. During this 11-month course, occurring 1 day per week, students practiced core clinical skills in inpatient and ambulatory patient care settings within four academic hospitals across Boston and their affiliated clinical sites. In these settings, students met patients, conducted interviews and physical examinations, and completed write-ups. Taught by approximately 200 clinical preceptors, approximately 140 medical students and 35 dental students completed the same course and curriculum each year. Three preceptors, each with a specific clinical skills focus, worked with each student throughout the year. These preceptors reviewed student notes, completed feedback forms on notes, and assessed students a few times during the year using EPAs and narrative evaluations.

Curriculum Implementation

The patient-centered documentation curriculum comprised a 45-minute asynchronous presession module, a 2-hour synchronous workshop during course time, and a practical application component throughout the year.

Asynchronous session: The 45-minute online asynchronous module (Appendix E) introduced students to the concept of open notes. This module reviewed the background of the 21st Century Cures Act, the history of open notes, interviews, and perspectives of patients familiar with open notes and key physician leaders in the field. Learners also reviewed the comprehensive student documentation guides (Appendices F and G) and completed a presession survey.

Synchronous workshop: Half the class on two different dates received a 2-hour, in-person workshop led by course faculty in the first month of the course. The workshop consisted of three main components: (1) an overview didactic session, (2) a student-driven brainstorming session and large-group discussion reviewing material from the asynchronous module, and (3) active case-based learning in small groups.

The workshop began with a 20-minute presentation delivered to the large group, providing an overview of the types and purpose of medical notes, the structure of a note, and how this curriculum integrated into the larger clinical skills course (Appendix H). After this brief didactic overview, we implemented active learning strategies to review the asynchronous session on open notes. Faculty divided the room in half to discuss perspectives and ethical quandaries of open notes, with one-half responsible for the patient point of view and the other for that of health care providers. Participants had 15 minutes to brainstorm in smaller groups of three to four students. The groups then reconvened for a 15-minute large-group discussion, with a reporter sharing from each group and the facilitator recording responses on a blank slide.

After the large-group session, students migrated to smaller classrooms with about 20 students in each. One facilitator was present per room to field questions and facilitate discussion. The facilitator distributed a sample case of an HPI (Appendix I). Students spent 10 minutes with a partner reading the case and highlighting areas done well and areas for improvement, with a focus on patient-centered language. The group then reconvened for 20 minutes, with each pair sharing its findings, thought processes, and reflections. The facilitator moderated the discussion. During the final 20 minutes, participants were introduced to the checklist of best practices and rubric with which future notes would be evaluated (Appendices A and B). The students utilized these to further cross-examine the sample note and practice rewriting different sections. The session ended with distribution of a revised model note for review at home (Appendix J).

Longitudinal practical application: After completion of the in-person workshop, students employed the strategies and skills they had learned throughout the remainder of the year, being responsible for writing 25 total notes based on real patient encounters. We directed faculty preceptors to use the patient-centered documentation rubric (Appendix B) on 15 total notes, five per clinical preceptor across the year. Faculty preceptors received the rubric via email through the Oasis platform at key time points during the 11-month course.

Assessment and Program Evaluation

We aimed to evaluate learners in three categories: attitude, knowledge, and skill as related to the Educational Objectives. We collected attitudinal data at two time points: in a survey immediately prior to curricular participation and in a formal course midyear evaluation survey 4 months after the main synchronous educational innovation, allowing learners to have time between learning the concepts in the workshop and practicing the skills in the clinical setting. Using a 5-point Likert scale (1 = *very unprepared/strongly disagree,* 5 = *very prepared/strongly agree*), we asked the same attitudinal questions in each survey (Appendix K) to measure how important participants felt patient-centered notes were for effective patient care, how prepared

Copyright © 2024 Eng et al. This is an open-access publication distributed under the terms of the Creative Commons Attribution license.

they felt to write clinical notes, and how prepared they felt to write patient-centered notes. Individual student responses were deidentified upon submission of surveys to Oasis. We performed statistical analysis through unpaired *t* tests comparing mean Likert-scale responses on the aggregate data. We considered *p* values $< .05$ statistically significant.

Within the midyear evaluation survey, students could optionally provide narrative comments about the curriculum and perceived effectiveness. We qualitatively evaluated these comments for themes. To assess the acquisition and sustainability of knowledge, we included questions within three mandatory written exams at different time points in the course. Each exam had three questions regarding this curricular content (Appendix L), which were self-authored (Anita Vanka and Katherine Johnston) and reviewed with the other authors and the course leadership. Directly derived from information and examples presented in the asynchronous module (Appendix E) and written documentation guide (Appendix G), these questions tested identification of person-first language, recognition of biased language, and understanding of open notes. We collected data for skills assessment in a subsequent project phase via preceptor evaluations of up to 15 notes for each student using the patient-centered documentation rubric.

Results

Of the 173 first-year students (138 medical and 35 dental) who participated, 172 responded to the preworkshop survey (response rate = 99%). All agreed that writing a patient-centered note was somewhat or very important for effective patient care (100%).

After completion of the synchronous workshop, 91 of 173 students completed the midyear course assessment (5 months after the synchronous session; response rate = 53%). As measured by mean Likert score (1 = *not prepared at all*, 5 = *very prepared*), students reported increased preparedness to write a clinical note (presession $M = 2.3$, midyear $M = 3.8$, $p < .001$) and to write a patient-centered note (presession $M = 2.2$, midyear $M = 3.9$, $p < .001$; Figure 1). Twenty-two students provided narrative comments. Thematic analysis revealed students felt the strengths of the curriculum lay in the early exposure, student-led discussions, and practical reinforcement of principles. The Table features student comments regarding barriers and improvements to the workshop.

Of three knowledge questions assessed throughout the year (Figure 2), the mean percentage correct increased for the question testing identification of person-first language (question 1: exam 1 = 62% correct, exam 2 = 87% correct, exam 3 = 88% correct). In addition, the questions testing identification of biased language and understanding of open notes showed acquired and sustained knowledge over the year (question 2: exam 1 = 86% correct, exam 2 = 84% correct, exam 3 = 93% correct; question 3: exam 1 = 96%, exam 2 = 97% correct, exam 3 = 96% correct).

On review of preceptor faculty use of the rubric, each student had up to 15 total notes assessed with the rubric, with an average of 12.9 completed rubrics received per student. Two hundred twenty instructors submitted 2,232 evaluations using the rubric, with an average of 10.2 rubrics submitted per instructor. As noted above, assessment of the rubric is outside the scope of this publication.

Discussion

In this new era of electronic medical record transparency, our curriculum, consisting of asynchronous prework, an interactive synchronous session, and longitudinal practical application, aims to address the notable gap in preparedness that new medical students feel when writing a clinical note, especially in a patient-centered manner. Based on an extensive literature review and responses from focus groups of medical students, residents, patients, physicians, and key leaders in the field of OpenNotes, we developed a curriculum for first-year medical students that prepares them to have notes read by patients. This foundational skills curriculum, while not comprehensive, introduces documentation as a key aspect of patient care while acknowledging the responsibility and implications that come with it. Our approach begins with first-year students, laying foundational principles from the start of training.

Overall, feedback from the first pilot year of the curriculum was positive, with learners commenting on the usefulness and necessity of the session, especially early on in their training. Attitudinal assessments revealed that after completion of the asynchronous work and interactive workshop, participants felt significantly more prepared to write a patient-centered note, achieving one of our main objectives.

Students offered valuable suggestions to enhance the session. For example, they suggested including more subtle and typical examples for the sample patient note. Participants also recommended spending more time addressing particularly sensitive areas of patient-centered language such as substance use, pediatrics, or mental health. While the asynchronous preparatory work included mention of these topics, the synchronous session did not formally address them due to time constraints. Importantly, participants also described observing

Copyright © 2024 Eng et al. This is an open-access publication distributed under the terms of the Creative Commons Attribution license.

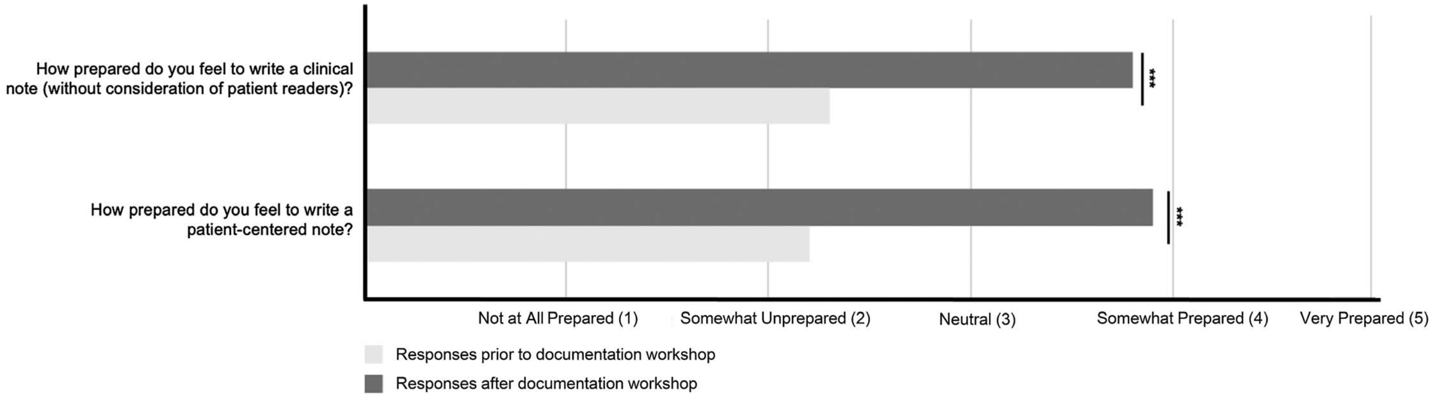

Figure 1. Results of attitudinal surveys before ($n = 172$) and after ($n = 91$) the documentation workshop. The three vertically stacked asterisks indicate statistical significance for all comparisons ($p < .001$).

discrepancies between the teaching and what attendings and residents practice. It is important instructors acknowledge this and emphasize that the skills they are teaching are part of an evolving change in best practices. Instructors may find it valuable to set aside time to address how to navigate observed discrepancies with superiors, an area of active development as part of our future curriculum for clerkship students.

From a facilitator perspective, we learned that, given this was the first introduction to notes for students, it was important,

Table. Themes From Open-Ended Midcourse Evaluations After Documentation Workshop ($N = 22$)

Theme	Analysis	Illustrative Comment
Most valuable aspects of workshop	• Valuable to learn these skills early in training<br>• Valuable to reinforce principles learned in future write-ups<br>• Students enjoyed discussion time in small groups	• “I really appreciate that we’re being taught about patient-centered documentation early in our training.”<br>• “Session made me more aware of language in notes… by putting ourselves in patient shoes.”<br>• “Helps that this is reinforced during the write-ups that we do.”<br>• “Liked the example we were given and that we got a chance to talk about it and how to improve in small groups.”
Barriers to applying lessons learned	• Students highlighted potential for discordance between behaviors taught in classrooms and behaviors witnessed in clinical practice<br>• Challenge for students to implement ideal approaches into evolving clinical practice environments<br>• Need for discussions on how to navigate these discrepancies	• “In this session, we learned how to not refer to patients in certain ways (e.g. Smoker) but then a week later, I saw an attending presenting his work on ‘Smokers’ and all the problems that come with that in his field. It would be great to have an explicit part of this on how we navigate making these language changes as medical students on the wards with superiors (who are responsible for evaluating us and our future opportunities) when situations like these arise.”<br>• “I have already encountered discrepancies between what was taught in this session and the expectations of my preceptors (specifically [Foundational Continuity Clinic]). I think it’s important to acknowledge that this model is not currently being used by the majority of doctors and that we will therefore need to be flexible in accommodating various preferences and styles.”
Improvements to workshop	• Recognition that generalized examples may not perfectly recapitulate the diverse array of challenges present in clinical settings<br>• Interest from students in exploring indication- and population-specific approaches to patient-centered documentation (e.g., substance use disorder)	• “I thought the example we used was too extreme. I wonder whether using a more typical but still problematic example would be better.”<br>• “I also think som[e] didactics around patient centered language (for instance, language around substance use) would be great.”<br>• “Wish we talked about how substance use ended up being important for the final diagnosis and how we could have balanced keeping the note patient-centered/de-stigmatizing for future providers while also ensuring all the important info was highlighted.”

Copyright © 2024 Eng et al. This is an open-access publication distributed under the terms of the Creative Commons Attribution license.

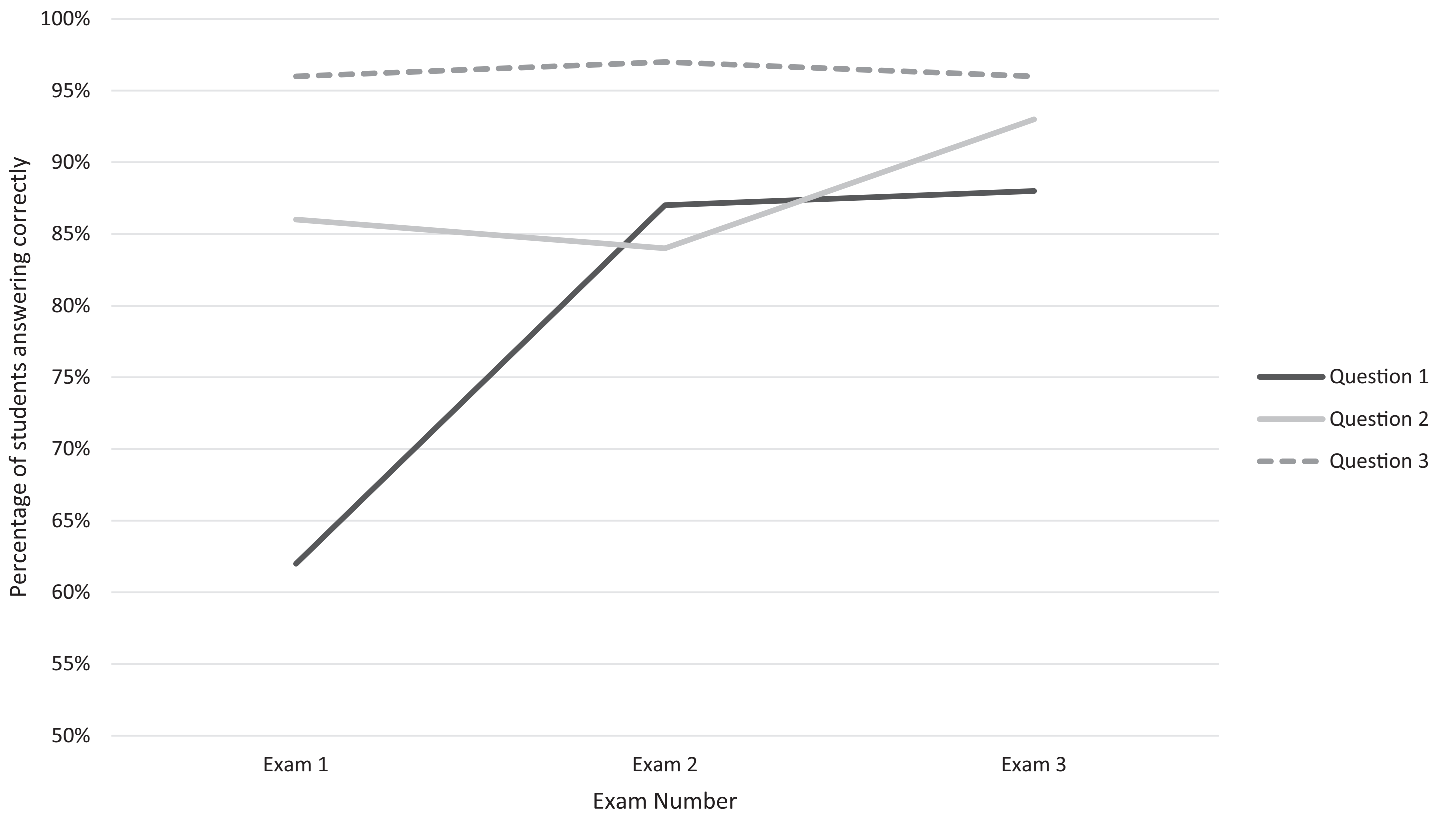

Figure 2. Results of three questions assessing knowledge tested in three exams throughout the year. Exam 1 was administered 2 months after the workshop (n = 173), exam 2 was administered 6 months after the workshop (n = 171), and exam 3 was administered 10 months after the workshop (n = 166). Question 1 assessed identification of person-first language, question 2 assessed recognition of biased language, and question 3 assessed understanding of open notes.

especially during the didactic, to stress that the focus of the session was on patient-centered language. Facilitators may not need to spend a large amount of time on the anatomy of the note (Appendix H) depending on when the specific sections are taught during a foundational clinical skills course. Facilitators should also anticipate having flexibility in timing during small groups, as the share-out discussion was often fruitful and required more time than the lesson plan allotted.

We acknowledge there are limitations to the curriculum and evaluation. A major limitation is that at the time of publication, we have not formally assessed the student notes and preceptor use of the rubric, affecting the full assessment of Educational Objective 3. Given the scope of resources and time needed to evaluate these, we will investigate this aspect in a subsequent project phase, allowing for a comprehensive analysis. Another limitation is that our evaluation of the curriculum relies on students' self-assessment and is thus vulnerable to subjective interpretation. Additionally, while almost all participants completed the precurriculum survey, fewer responded to the subsequent midyear course evaluation. We felt it appropriate to obtain our postworkshop attitudinal assessment within this midyear course evaluation to enhance participation and provide time to implement skills learned. However, this resulted in responses collected 4 months after the synchronous session and subject to recall bias. This also meant we could only collect aggregated data and not assign personal study IDs, preventing pairwise comparisons and use of paired t test analysis. These questions were also unable to be included in the end-of-year course evaluation (6 months after the midcourse evaluation), limiting assessment of student attitudes at the end of the course. Moreover, the knowledge-based assessment included only three questions on each examination, questions that were self-authored and reviewed among the authors of this study and course leadership. More extensive and validated knowledge assessments would likely provide additional data, such as demonstrating change in the knowledge of these concepts, augmenting our assessment of Educational Objectives 1 and 2. Lastly, given that this curriculum is only in place for first-year students, we cannot prove it leads to sustained skill changes,

Copyright © 2024 Eng et al. This is an open-access publication distributed under the terms of the Creative Commons Attribution license.

especially in stages of training with novel practical demands and time constraints.

Next steps include evaluating the efficacy of this curriculum on skills by assessing the student-written clinical notes and validating the newly developed rubric. Once validated, an expanded curriculum can reach other levels of learning, including clerkship students, postclerkship students, residents, and faculty teachers, and promote a more global shift in documentation practices.

In summary, this novel curriculum utilizes a multipart approach to prepare the next generation of learners to employ clinical notes as a tool to communicate with patients and providers, with special attention to how readers receive a note. The curriculum introduces key ethical considerations and points out how documentation can enhance or harm therapeutic relationships with patients. While the longitudinal impact remains to be seen, it is clear patient-centered documentation skills should be an integral part of documentation education.

Appendices

A. Checklist of Best Practices.docx
B. Rubric.docx
C. Facilitator Guide.docx
D. Course Planner Implementation Guide.docx
E. Asynchronous Module folder
F. Student Guide.docx
G. Written Documentation Guide.docx
H. Student Session Slides.pptx
I. Sample Note.docx
J. Model Note.docx
K. Attitudinal Survey Questions.docx
L. Knowledge Questions.docx

All appendices are peer reviewed as integral parts of the Original Publication.

Kathleen Eng: Fourth-Year Medical Student, Harvard Medical School; ORCID: https://orcid.org/0000-0001-7012-9641

Katherine Johnston, MD: Assistant Professor, Department of Medicine, Harvard Medical School; ORCID: https://orcid.org/0000-0002-4543-2419

Ivo Cerda, MSE: Third-Year Medical Student, Harvard Medical School; ORCID: https://orcid.org/0000-0003-4992-9841

Kushal Kadakia, MSE, MSt: Third-Year Medical Student, Harvard Medical School

Alison Mosier-Mills: Fourth-Year Medical Student, Harvard Medical School

Anita Vanka, MD: Assistant Professor, Department of Medicine, Harvard Medical School; ORCID: https://orcid.org/0000-0001-6536-6925

Disclosures

None to report.

Funding/Support

Dr. Anita Vanka received funding from the Josiah Macy Jr. Foundation.

Prior Presentations

Eng K, Johnston K, Vanka A. Say what you'll write, write what you say: a patient-centered documentation skills curriculum in the era of patient-accessible notes. Poster presented at: Society of General Internal Medicine Annual Meeting; May 12, 2023; Aurora, CO.

Eng K, Vanka A, Johnston K. Say what you'll write, write what you say: teaching patient-centered documentation skills. Presented at: Society of General Internal Medicine New England Regional Meeting; September 17, 2022; Boston, MA.

Ethical Approval

Reported as not applicable.

References

1. 21st Century Cures Act, Pub L No. 114-255, 130 Stat 1033 (2015).
2. Nazi KM, Turvey CL, Klein DM, Hogan TP, Woods SS. VA OpenNotes: exploring the experiences of early patient adopters with access to clinical notes. *J Am Med Inform Assoc*. 2015; 22(2):380-389. https://doi.org/10.1136/amiajnl-2014-003144
3. Walker J, Leveille S, Bell S, et al. OpenNotes after 7 years: patient experiences with ongoing access to their clinicians' outpatient visit notes. *J Med Internet Res*. 2019;21(5):e13876. https://doi.org/10.2196/13876
4. Delbanco T, Walker J, Bell SK, et al. Inviting patients to read their doctors' notes: a quasi-experimental study and a look ahead. *Ann Intern Med*. 2012;157(7):461-470. https://doi.org/10.7326/0003-4819-157-7-201210020-00002
5. Crotty BH, Anselmo M, Clarke D, et al. Open notes in teaching clinics: a multisite survey of residents to identify anticipated attitudes and guidance for programs. *J Grad Med Educ*. 2018; 10(3):292-300. https://doi.org/10.4300/JGME-D-17-00486.1
6. Shen E, Cristiano JA, Ellis LR. The electronic health record objective structured clinical examination station: assessing student competency in patient notes and patient interaction.

Copyright © 2024 Eng et al. This is an open-access publication distributed under the terms of the Creative Commons Attribution license.

MedEdPORTAL. 2020;16:10998. https://doi.org/10.15766/mep_2374-8265.10998

7. Beach MC, Saha S, Park J, et al. Testimonial injustice: linguistic bias in the medical records of Black patients and women. *J Gen Intern Med*. 2021;36(6):1708-1714. https://doi.org/10.1007/s11606-021-06682-z
8. Kelly JF, Dow SJ, Westerhoff C. Does our choice of substance-related terms influence perceptions of treatment need? An empirical investigation with two commonly used terms. *J Drug Issues*. 2010;40(4):805-818. https://doi.org/10.1177/002204261004000403
9. Blease C, Torous J, Hägglund M. Does patient access to clinical notes change documentation? *Front Public Health*. 2020;8:577896. https://doi.org/10.3389/fpubh.2020.577896
10. Ralston JD, Yu O, Penfold RB, Gundersen G, Ramaprasan A, Schartz EM. Changes in clinician attitudes toward sharing visit notes: surveys pre- and post-implementation. *J Gen Intern Med*. 2021;36(11):3330-3336. https://doi.org/10.1007/s11606-021-06729-1
11. Amiel J, Ryan MS, Andriole DA, Whelan AJ. *Core Entrustable Professional Activities for Entering Residency: Summary of the 10-School Pilot, 2014-2021*. Association of American Medical Colleges; 2022. Accessed February 13, 2024. https://store.aamc.org/downloadable/download/sample/sample_id/581/
12. Patel A, Ali A, Lutfi F, Nwosu-Iheme A, Markham MJ. An interactive multimodality curriculum teaching medicine residents about oncologic documentation and billing. *MedEdPORTAL*. 2018;14:10746. https://doi.org/10.15766/mep_2374-8265.10746
13. Lai J, Tillman D. Curriculum to develop documentation proficiency among medical students in an emergency medicine clerkship. *MedEdPORTAL*. 2021;17:11194. https://doi.org/10.15766/mep_2374-8265.11194
14. Skelly K, Shen W, Wilbur J, et al. A curriculum for teaching clinical efficiency focusing on specific communication skills while maximizing the electronic health record. *MedEdPORTAL*. 2020;16:10989. https://doi.org/10.15766/mep_2374-8265.10989
15. Zavodnick J, Kouvatsos T. Electronic health record skills workshop for medical students. *MedEdPORTAL*. 2019;15:10849. https://doi.org/10.15766/mep_2374-8265.10849
16. Bynum D, Colford C, McNeely D. Writer's workshop: teaching preclinical medical students the art of the patient "write-up." *MedEdPORTAL*. 2014;10:9805. https://doi.org/10.15766/mep_2374-8265.9805
17. Stagno S, Crapanzano K, Schwartz A. Keeping the patient at the center: teaching about elements of patient-centered care. *MedEdPORTAL*. 2016;12:10500. https://doi.org/10.15766/mep_2374-8265.10500
18. Raney J, Pal R, Lee T, et al. Words matter: an antibias workshop for health care professionals to reduce stigmatizing language. *MedEdPORTAL*. 2021;17:11115. https://doi.org/10.15766/mep_2374-8265.11115
19. Wilson LO. *Anderson and Krathwohl: Bloom's Taxonomy Revised—Understanding the New Version of Bloom's Taxonomy*. Quincy College; 2016. Accessed February 13, 2024. https://quincycollege.edu/wp-content/uploads/Anderson-and-Krathwohl_Revised-Blooms-Taxonomy.pdf
20. Graffam B. Active learning in medical education: strategies for beginning implementation. *Med Teach*. 2007;29(1):38-42. https://doi.org/10.1080/01421590601176398
21. Baker EA, Ledford CH, Fogg L, Way DP, Park YS. The IDEA assessment tool: assessing the reporting, diagnostic reasoning, and decision-making skills demonstrated in medical students' hospital admission notes. *Teach Learn Med*. 2015;27(2):163-173. https://doi.org/10.1080/10401334.2015.1011654

Received: August 28, 2023
Accepted: January 4, 2024
Published: March 26, 2024

Copyright © 2024 Eng et al. This is an open-access publication distributed under the terms of the Creative Commons Attribution license.
