# Supplementary material for: A Patient-Centered Documentation Skills Curriculum for Preclerkship Medical Students in an Open Notes Era
Source: MedEdPORTAL. 2024 Mar 26;20:11392. doi: 10.15766/mep_2374-8265.11392 (PMC10963659; doi:10.15766/mep_2374-8265.11392)
Supplement: Supplementary file 1 — Checklist of Best Practices.docxRubric.docxFacilitator Guide.docxCourse Planner Implementation Guide.docxAsynchronous Module folderStudent Guide.docxWritten Documentation Guide.docxStudent Session Slides.pptxSample Note.docxModel Note.docxAttitudinal Survey Questions.docxKnowledge Questions.docx [file mep_2374-8265.11392-s001.zip › A. Checklist of Best Practices.docx]

*Appendix A: Checklist of Best Practices*

**Checklist of Best Practices for Patient-Centered Documentation**

- Use person-first language.
- Refer to your patient as how they want to be identified.
- Avoid abbreviations and acronyms, especially if not officially approved by the hospital.
- Say what you write, write what you say!
- Verify past history information before including in the note.
- Avoid words which can confer bias and judgment.
- Keep physical exam descriptions objective.
- Empower your patients with encouraging words and clear next steps.
- Pay close attention to sensitive topics, including but not limited to sexual history, trauma history, substance history, mental health history.
- Write from your perspective.
