# Supplementary material for: A Patient-Centered Documentation Skills Curriculum for Preclerkship Medical Students in an Open Notes Era
Source: MedEdPORTAL. 2024 Mar 26;20:11392. doi: 10.15766/mep_2374-8265.11392 (PMC10963659; doi:10.15766/mep_2374-8265.11392)
Supplement: Supplementary file 1 — Checklist of Best Practices.docxRubric.docxFacilitator Guide.docxCourse Planner Implementation Guide.docxAsynchronous Module folderStudent Guide.docxWritten Documentation Guide.docxStudent Session Slides.pptxSample Note.docxModel Note.docxAttitudinal Survey Questions.docxKnowledge Questions.docx [file mep_2374-8265.11392-s001.zip › B. Rubric.docx]

*Appendix B: Rubric*

**Patient-Centered Documentation Assessment Rubric**

Write up number (select number between 1-14):

Date of encounter:

Instructions: Rate the quality of each section of the written note based on the extent to which defined elements are present

**1 =** minimal to no elements (early)

**2 =** some to many elements (good)

**3 =** most or all elements (excellent)

**NA** – not applicable

|  | **1** | **2** | **3** | **N/A** |
| --- | --- | --- | --- | --- |
|  | **None/minimal** | **Some** | **Complete** |  |
| **WRITTEN HISTORY** | | | | |
| **Detailed HPI**  Defined as including a completed description of the chief concern (s) such as location, quality, severity, duration, timing, radiation, factors that aggravate or alleviate symptoms |  |  |  |  |
| **Descriptive HPI**  Defined as use of semantic and descriptive vocabulary such as acute or chronic, sharp or dull, continuous or intermittent |  |  |  |  |
| **Chronologic HPI**  Defined as telling a clear story that flows logically |  |  |  |  |
| **Contextualized HPI**  Defined as identification and inclusion of key findings from past, family, and social history and relevant other symptoms that might otherwise belong in later portions of the comprehensive history |  |  |  |  |
| **Complete Comprehensive History**  Defined as complete and verified past medical, surgical, social and family history, and complete review of systems |  |  |  |  |
| **WRITTEN PHYSICAL EXAM FINDINGS** | | | | |
| **Complete Physical Examination**  Defined as including all elements of the foundational head-to-toe exam performed during the patient encounter |  |  |  |  |
| **Key Physical Exam Findings**  Defined as including the hypothesis-directed pertinent exam findings based on the presenting chief concern(s) |  |  |  |  |
| **PATIENT-CENTERED DOCUMENTATION** | | | | |
| **Patient-Centered Description**  Defined as referring to the patient as they want to be described, using person-first language and social history   - Honorifics, gender pronouns, individual attributes - Patient is an individual *with* the condition, not *defined by* the condition |  |  |  |  |
| **Patient-Centered Language**  Defined as avoiding acronyms and words or phrases that connote bias, contribute to judgments, and propagate misunderstandings of diseases   - Deliberate use of judgment-free verbs (i.e., “concern” instead of “complaint”; “reports” or “says” or “tells”, instead of “denies”, “endorses”, “claims”) - Avoidance of terms which can confer bias (i.e., “compliance”, “adherence”, “AMA”) - Exclusion of acronyms (i.e., “SOB”, “iso”, “f/u”, “r/o”, “MOP”) - Thoughtful & judicious use of quotation marks - Avoidance of language which can be considered coded (i.e., “poor historian”, “patient is quite involved in their care”, any judgements about explanatory models) |  |  |  |  |

Optional Narrative Feedback:

Optional open text box

Modified from:

Baker EA, Ledford CH, Fogg L, Way DP, Park YS. The IDEA Assessment Tool: Assessing the Reporting, Diagnostic Reasoning, and Decision-Making Skills Demonstrated in Medical Students’ Hospital Admission Notes. *Teach Learn Med*. 2015;27(2):163-173. doi:10.1080/10401334.2015.1011654
