# Supplementary material for: A Patient-Centered Documentation Skills Curriculum for Preclerkship Medical Students in an Open Notes Era
Source: MedEdPORTAL. 2024 Mar 26;20:11392. doi: 10.15766/mep_2374-8265.11392 (PMC10963659; doi:10.15766/mep_2374-8265.11392)
Supplement: Supplementary file 1 — Checklist of Best Practices.docxRubric.docxFacilitator Guide.docxCourse Planner Implementation Guide.docxAsynchronous Module folderStudent Guide.docxWritten Documentation Guide.docxStudent Session Slides.pptxSample Note.docxModel Note.docxAttitudinal Survey Questions.docxKnowledge Questions.docx [file mep_2374-8265.11392-s001.zip › C. Facilitator Guide.docx]

*Appendix C: Facilitator Guide*

**A Patient-Centered Documentation Skills Curriculum for Pre-Clerkship Medical Students in an Open Notes Era**

**Learning Objectives:**

1. Define the 21^st^ Century Cures Act and the research of OpenNotes and patient engagement.
2. Identify language used in discussing patients and documenting patient concerns that could be harmful to patients, create unwanted bias, or adversely affect other health professionals’ understanding of patients.
3. Demonstrate a patient-centered approach to documentation and increase self-reported preparedness in using nondiscriminatory, non-judgmental, and inclusive language in clinical notes.

**Pre-Session Preparation:**

In addition to this Facilitator guide, facilitators should also review the following:

1. **Student session slides** (Appendix H)
2. **Written Documentation Guide** (Appendix G)

**Agenda:**

| **Estimated duration** | **Session Component** | **Page #** |
| --- | --- | --- |
| **50 minutes** | **Part 1: Large Group Session** | **2 - 7** |
| - 20 minutes | Overview of Clinical Notes | 2- 6 |
| - 30 minutes | Discussion: Takeaways from Asynchronous Session on Open Notes | 6 - 8 |
| **10 minutes** | **Break and transition into small groups** |  |
| **60 minutes** | **Part 2: Small Group Session** | **8 - 10** |
| - 35 minutes | Introduction & Dissecting Prepared Note | 8 - 9 |
| - 25 minutes | Introduce Best Practices Checklist & Rewrite Note Using Rubric | 9 - 10 |

**Learning Patient-Centered Documentation Skills**

**Detailed Agenda:**

**Part 1: Large Group Session (50 minutes total)**

Session objectives

- Provide an overview of clinical notes, including their purpose, components, and types of notes.
- Discuss takeaways from asynchronous preassigned module on patient-centered documentation.
- Introduce checklist of best practices for patient-centered documentation.

The Student Session Slides (Appendix H) accompany Part 1. Please refer to this PowerPoint presentation for further details. Slide numbers corresponding to the section subheadings are indicated here.

- 1. **Overview of Clinical Notes (20 minutes)**
     1. Introduction (slides 1 and 2)

Introduce yourself and other facilitators. Provide an overview of the morning, emphasizing the two components of this synchronous session (large group and small group discussion).

- - 1. What is the purpose of the clinical note? (slide 3)

You may start the discussion by asking students to share ideas about the different purposes of the clinical note as a way to stimulate participation and critical thinking. Depending on the level of training, students may be prompted to share their own experience with notes as patients and/or care partners. After acknowledging a few contributions, you may share the following purposes of the written clinical note:

The written note serves as a…

1. record of the encounter with the patient
2. platform to express your thoughts and recommendations
3. mechanism to share information with the patient and other healthcare professionals involved in the patient’s care

Here is a good point to **introduce the Cures Act and the background work of OpenNotes preceding this federal mandate.**

Since April 2021, new federal rules mandate that all patients (with several permitted exceptions) are offered rapid, online access to their clinical records, including the notes written by clinicians (‘open notes’). Extensive survey research demonstrates that patients who review their records and read their notes feel more involved in and knowledgeable about their care, better prepared for visits, and report being more likely to follow their clinicians’ advice.

- - 1. What are the different types of clinical notes? (slide 4)

You may begin by posing the question to the group and allowing for brief responses. After acknowledging the different answers, you may share that written notes taught in introductory clinical courses usually focus on the history and physical note (also known as the “H&P” note). For students in their early clinical training, these notes will describe encounters with patients who are seen for an initial visit, follow-up visit, or urgent care visit in the office setting; and with patients who are hospitalized for a specific reason.

The H&P note acts as basis for many other note types (like hospital admission note, ER visit note, etc.) but components may be expanded on or truncated depending on the situation. For example, a full history and physical is required for a new patient visiting the hospital or an office, whereas progress or follow-up notes may be more abbreviated and focused.

Other types of notes include telephone or communications note, telemedicine visit note, ER visit note, brief and complete operative reports, and discharge summaries.

- - 1. What are the sections that make up the anatomy of a note? (slides 4 and 5)

At this time, you can review that the clinical note is broken down into components that systematically build towards the patient’s possible diagnosis and recommendations for next steps in diagnostic workup and management.

The note begins with a description of what is bringing the patient to medical attention, followed by a more thorough description of their symptomatology and additional background medical information.

- Chief Concern (CC): the writer needs to capture one or two main concerns that brought the patient to seek medical attention. These symptoms will make the case for a likely diagnosis, but do not represent the diagnosis itself.
  - For example, CC would be “chest pain,” and not myocardial infarction or pulmonary embolism.
- One-liner: this sentence attempts to capture *who* the patient is. It includes *pertinent* past medical history and risk factors, the main symptoms bringing the patient to seek medical attention (i.e., chief concern), the temporal pattern of these symptoms (using descriptive terms such as “progressive”, or numerical terms such as “1 week”)
- History of Present Illness (HPI): usually a number of paragraphs that tell the story surrounding the patient’s chief concern. It weaves the information provided by the patient into a coherent narrative that includes a chronological description of the manifestations of illness, attempted interventions, and patient interpretations. The mnemonic “OPQRSTAA” might be helpful to further characterize a concern related to pain or discomfort.
- Review of Systems (ROS): this is a comprehensive list of closed-ended questions organized by system. There are 14 recognized systems.

A comprehensive and complete report of the patient’s background is also included. It usually includes past medical history, past surgical history, medications, allergies and adverse reactions, family history, and social history.

Then, an objective assessment of the patient is noted – it usually includes a report of the physical exam and relevant laboratory and test results.

- Physical Exam (PE), tailored to CC: this section documents the vital signs and the foundational physical exam, as well as the comprehensive exam for the organ systems pertinent to the chief concern.

The note ends with a summary, assessment, and plan of the patient’s presenting concern, which addresses all pertinent findings on the physical exam and other data, and initial diagnostic considerations.

- Summary: this sentence should integrate key data you collected, preparing you to argue what you think diagnosis is. It is not the opening statement in the HPI.
- Assessment: This is a summative statement that argues what you think the diagnosis is and how you explain: “Why this patient, why now?”
- Plan: This is your recommendation for each problem identified for the patient, organized by differentials being considered for this problem/symptom, and in order of importance for each problem.
  - Within it, you should include a differential diagnosis, diagnostic plan, and therapeutic interventions. Please note that chronic problems do not need a fleshed out differential.

Next, please introduce and highlight the **Written Documentation Guide (Appendix G),** which students should have read as part of their preassigned work. Please emphasize that this guide includes thorough explanations for each component of the clinical note, and relevant examples.

- - 1. Introduction of best practices and rubric (slides 7, 8, and 9)

Please introduce the checklist of best practices (Appendix A) and highlight each of the 10 items with specific examples of each principle. These are:

- Use person-first language.
- Refer to your patient as how they want to be identified.
- Avoid abbreviations and acronyms, especially if not officially approved by the hospital.
- Say what you write, write what you say.
- Verify past history information before including in the note.
- Avoid words which can confer bias and judgment.
- Keep physical exam descriptions objective.
- Empower your patients with encouraging words and clear next steps.
- Pay close attention to sensitive topics, including but not limited to sexual history, trauma history, substance history, mental health history.
- Write from your perspective.

Next, please highlight the rubric schematic (Appendix B), emphasizing that it incorporates the best practices introduced in the previous slide. Students can refer to this rubric when writing and reviewing their notes.

Additionally, an optional component is to describe the process by which the checklist and rubric were developed. We highly recommend taking the time to describe the process, as it allows for transparency and offers an opportunity to build credibility.

The checklist of best practices and rubric were developed originally by Harvard Medical School researchers, educators, and students through…

- Literature review and focus groups with key leaders in the field.
- Key literature from Open Notes and medical education initiatives.
- Focus groups with medical students, internal medicine residents, patient advocates and experts in Open Notes, physician experts in Open Notes, and medical student educators.
  - The standard questions were developed by researchers specializing in medical education and approved by IRBs from Beth Israel Deaconess Medical Center, Massachusetts General Hospital, and Harvard Medical School.
  - A thematic analysis of the responses was carried out by key investigators in the field.
- Finally, different iterations of the checklist and grading rubric were shared with medical student educators for feedback, pilot, and iteration.
  - 1. What will students learn regarding clinical notes during the early phases of their clinical training? (slides 10, 11, and 12)

First, please discuss the importance of a curriculum on patient-centered documentation. You may or may not pose the following question to the group: “what are some potential benefits of learning about patient-centered documentation?’

A curriculum on patient-centered documentation focused on medical students early in their clinical training is necessary and beneficial given that:

- In the era of OpenNotes, electronic medical record (EMR) transparency is the new normal and we are all learners, including faculty and residents who have been writing clinical notes for a number of years.
- Patient-centered documentation ultimately benefits patients, especially vulnerable populations, who are at higher risk for the deleterious effects of stigmatizing language and documentation.
- Patient-centered documentation strengthens the therapeutic alliance with patients and empowers patients to partner with the clinician in their care.
- It is important to learn how to write patient-centered notes from the beginning, to create and reinforce good habits that will persist throughout later stages of clinical training.

This is good segue into expectations for students early in their clinical training as they learn about documentation. Students should expect to:

- Identify language used in discussing patients and documenting patient concerns that could be harmful to patients, create unwanted bias, or adversely affect other health professionals’ understanding of patients.
- Drawing on a specified checklist for guidance, demonstrate a patient-centered approach to documentation, using nondiscriminatory, non-judgmental, and inclusive language.
- Reflect on clinical documentation with patients, hear the perspective of the patient, and receive feedback on whether the student effectively employed an engaging, patient-centered approach to documentation.

Next, institutions are encouraged to inform their students of the different instances in their respective curriculums during which students will learn oral presentation and documentation skills. It is helpful to highlight where and how this session and its accompanying asynchronous module fit in the larger curriculum specific to each school.

- 1. **Discussion: Takeaways from Asynchronous Session on Open Notes (30 minutes)**
     1. Introduction (slide 13)

The facilitator is encouraged to invite students to recap the Asynchronous module (Appendix E) with the goal of coming to a shared understanding of the benefits of transparent notes to patients, healthcare professionals, and some of the ethical dilemmas this may pose. This is a good time to learn about the students’ personal reflections and experiences as they relate to OpenNotes and share insights that students themselves developed from the asynchronous module.

- - 1. Breakout discussion (slide 14) – 10 minutes

In this breakout session, the facilitator should devise a simple way to assign questions to different groups. One such alternative is assigning questions according to location in the room (i.e., left side of the room answers A, middle of the room answers B, right side answers C)

Introduce the exercise using the following prompts:

- Please turn to your nearest neighbors and form groups of 3 or 4
- Once the groups have been formed:
  - Introduce yourself.
  - Assign a reporter for each group.
  - Discuss one of the following questions:

1. How might patient-centered documentation **benefit patients**?
2. How might patient-centered documentation **benefit healthcare professionals**?
3. What are some **potential ethical quandaries** that might arise with patient-centered and shared notes?
   - Each group should be prepared to report out to the larger group.
   - Regroup in 10 minutes.
     - At that point, a visible timer might be started.

- Some key points for each question to guide discussion include:
  - How might patient-centered documentation benefit patients?
    - Open notes enhance communication for patients and their families.
    - Open notes reduce barriers for caregivers and non-English speaking patients, especially in managing their health and coordinating care.
    - Referring to notes can assist patients with recalling visit details.
  - How might patient-centered documentation benefit healthcare professionals?
    - Open notes augment trust and therapeutic alliances between patients and health care professionals.
    - Open notes can reduce medical errors and enhance patient safety.
    - Open notes can enhance the partnership between physicians and patients in the patient’s medical care.
  - What are some potential ethical quandaries that might arise with patient-centered and shared notes?
    - Reflection is needed when considering sensitive issues: privacy considerations, gender identity, sexual health, trauma experiences, substance use, mental health history.
    - Barriers exist for patient engagement with open notes such as differences in digital access and language preferences.
    - Patient-centered language is critical in an open notes era; otherwise, the potential of stigmatizing and biased language to perpetuate clinician bias and healthcare disparities is even more enhanced when patients can read their notes.
    - Improper documentation practices may jeopardize the physician-patient relationship.
    - Open notes can affect how a physician may document the visit, potentially affecting accuracy and quality of the note.
    1. Large group share-out (slides 15 and 16) – 20 minutes

Groups are invited to share via the assigned reporter. The facilitator is encouraged to write down themes from each group on to the board or slide, so that everyone in the room can see and hear what is being shared. If more than one facilitator is present, one can moderate the discussion while the other takes notes.

A summary slide reinforcing best practices introduced earlier as a checklist and embedded in the rubric can be recapped once the discussion has finished.

- - 1. Transition to small group activity (slides 16, 17, 18, and 19) – 10-minute break

Having presented an overview of clinical notes, shared lessons from the asynchronous module, and presented and reinforced a summary of best practices, it is now time to delve deeper by working in small groups.

First, we will simulate a patient encounter and dissect a pre-written clinical note of the encounter. Then, we will review the note using the rubric based on best practices and attempt to rewrite part of the note informed by the principles of patient-centered documentation explored in this session.

Students are reminded of the following:

- Physical location of small group discussions (if in-person)
- Where they can find their group assignment (we recommend sharing a list with pre-assigned groups with students ahead of time to minimize confusion)
- Start time (we recommend 10 minutes after the end of Part 1)

We recommend providing contact information of facilitators or educators in charge of the implementation of the documentation curriculum in case students have questions, comments, or feedback.

**Part 2: Small Group Session (60 minutes total)**

Session objectives

- Identify language used in discussing patients and documenting patient concerns that could be harmful to patients, create unwanted bias, or adversely affect other health professionals’ understanding of patients.
- Demonstrate a patient-centered approach to documentation, using nondiscriminatory, non-judgmental, and inclusive language, using a specified checklist as guidance.

Materials needed:

- Sample note (HPI only) – one copy per student (Appendix I)
- Checklist of best practices – one copy per student (Appendix A)
- Rubric – one copy per student (Appendix B)
- Model note (HPI only) – one copy per student (Appendix J)
  1. **Introduction & Dissecting Prepared Note (35 minutes)**
     1. Introduction (5minutes)

Introduce yourself and other facilitators, if present. Depending on the size of the group, you might want people to introduce themselves. Consider an “icebreaker” to facilitate participation and open collaboration. At this point, you may introduce the session objectives (see above).

Please hand out copies of the Sample Note (Appendix I), which only contains the history of present illness. In addition to the hard copy, you may choose to put the sample note up in a screen, if available. Give everyone a few minutes to read the sample note on their own.

- - 1. Small group breakouts (15 minutes)

Introduce the small group breakout exercise using the following prompts:

- Please turn to your nearest neighbors and form groups of 3 or 4.
- Once the groups have been formed, discuss the following:
  - What was done well in this note?
  - What could be changed?
  - Why should some of those changes be implemented?
- You have 15 minutes to discuss.
- Each group should be prepared to report out to the larger group.
  - 1. Share out (10 minutes)

The facilitator should now encourage sharing and reflection of what was discussed in the small groups. Options include having each breakout group comment on a specific portion of the note or engage in open discussion. It is encouraged to prompt learner reflections about why some of these sentences should be changed. For example, consider the following questions:

- How can some of these sentences affect the patient if they were to read them?
- How would some of these sentences negatively affect patient care?
- Why can some of these sentences adversely affect the patient?
- Why can some of these sentences affect perceptions of other clinicians reading the note?

Some key features of the sample note that should be identified and discussed as areas of non-patient centered language include:

- “Chief Complaint” is better referred to as “Chief Concern”.
- Use person-first language: for example, replacing “52 y.o. IV drug user” with “patient with a history of IV drug use)
- Avoid words that suggest disbelief, suggest a negative tone, or can carry negative connotation such as “insists”, “denies”, “claims”, “complains”, “alleges”, “leaving AMA even though he was well aware of the risks,” “poor control”, “unmotivated”, “refused”.
- Avoid terms that confer bias and can perpetuate negative stereotypes such as “drug of choice”, “dirty”, “likes to lick needles”, “drug habit”. Instead, use terms that focus on the medical nature of substance use disorder and treatments.
- Be judicious with use of quotes, avoiding using them in a way that suggests disbelief.
- Use more detail and descriptors to characterize the chief concern (detailed and descriptive HPI)
  - Unanswered questions that might remain in the reader’s mind include: Which leg is involved? Which part of the back is affected? Are there better descriptors to describe quality and severity? What makes his symptoms better or worse? Exactly how many days ago did this start? Is the pain constant or intermittent?
- Reorder details to improve its logical flow and tell a clear story that flows logically (chronologic HPI). May suggest initially confusing on chief concern and the progression of symptoms prior to giving relevant background.
- Consider whether the one-liner should and needs to include the substance history
  - What are the implications when this is included upfront?
  - What might the reader or writer be thinking when including this in the one-liner?
  - What information should be in the one-liner?
- Consider whether the HPI needs to include the degree of focus on substance use that it currently contains.
  - Is this because the writer feels the back pain relates to the substance use?
  - Should this information be in other parts of the history instead, such as the Past Medical History?
  - How might our perception of the patient be affected with this information upfront in the HPI vs. placing these details later in the complete history, such as in the Past Medical History?
  1. **Introduce Checklist of Best Practices & Rewrite Note Using Rubric (25 minutes)**
     1. Re-introduction of Checklist of Best Practices and Rubric (5 minutes)

Please hand out copies of the Checklist of Best Practices (Appendix A) and Rubric (Appendix B). Remind students of the best practices for patient-centered documentation introduced in the large session. You may want to put up the Best Practices Checklist on a screen, if available, as you do this. These best practices are included again below. The facilitator is encouraged to review these best practices with specific examples.

- Use person-first language.
- Refer to your patient as how they want to be identified.
- Avoid abbreviations and acronyms, especially if not officially approved by the hospital.
- Say what you write, write what you say.
- Verify past history information before including in the note.
- Avoid words which can confer bias and judgment.
- Keep physical exam descriptions objective.
- Empower your patients with encouraging words and clear next steps.
- Pay close attention to sensitive topics, including but not limited to sexual history, trauma history, substance history, mental health history.
- Write from your perspective.

Re-introduce the Rubric mentioned in the large group session. Remind students that they can refer to this rubric when writing and reviewing their notes. Similarly, you might want to put the rubric up on a screen, if available.

- - 1. Re-writing note using the rubric (15 minutes)

Please assign each small breakout group to one portion or paragraph of the sample note. The number of breakout groups will dictate the specific breakdown of the note.

Once the groups are assigned, you may use the following prompts to introduce the next brief exercise:

- Please reform your previous groups of 3 or 4.
- Re-write your assigned portion or paragraph of the note with the checklist of best practices we have discussed and with the rubric in mind.
- You have 15 minutes.
  - 1. Hand out Model Note (5 minutes)

Once the groups have finished, please hand out copies of the Model Note (Appendix J). Students should take the model note home. Encourage them to review it and compare it to their attempt. If time allows, you might model that review by briefly pointing out some of the key changes highlighted in the model note.

**Conclusion**

In the era of open notes, it is vital to be cognizant of how we represent our patients and the clinical encounter in written. Although open notes address an important ethical issue - ensuring that patients have access to what is written by clinicians in their record - they also present novel ethical and practice dilemmas. Words matter, now more than ever. By mentioning social determinants of health that further contextualize a patient’s clinical presentation, avoiding words that can confer and perpetuate bias and judgment, and including encouraging statements we can empower our patients, positively impact their clinical course, and facilitate healing.
