# Supplementary material for: A Patient-Centered Documentation Skills Curriculum for Preclerkship Medical Students in an Open Notes Era
Source: MedEdPORTAL. 2024 Mar 26;20:11392. doi: 10.15766/mep_2374-8265.11392 (PMC10963659; doi:10.15766/mep_2374-8265.11392)
Supplement: Supplementary file 1 — Checklist of Best Practices.docxRubric.docxFacilitator Guide.docxCourse Planner Implementation Guide.docxAsynchronous Module folderStudent Guide.docxWritten Documentation Guide.docxStudent Session Slides.pptxSample Note.docxModel Note.docxAttitudinal Survey Questions.docxKnowledge Questions.docx [file mep_2374-8265.11392-s001.zip › D. Course Planner Implementation Guide.docx]

*Appendix D: Course Planner Implementation Guide*

**A Patient-Centered Documentation Skills Curriculum for Pre-Clerkship Medical Students in an Open Notes Era**

**>6 months prior to session: Identify session setting and timing**

Begin by determining an appropriate time to conduct this workshop, which will vary based on the specifics of your school's curriculum. At our institution, this session took place within a larger initiative to educate students and instructors about shared medical records (OpenNotes). Broadly, the initiatives include one faculty development and one student workshop incorporated into the pre-clerkship year into the foundational clinical skills course.

We found that early introduction of this concept was helpful in establishing patient-centered documentation as a foundational skill for the practice of medicine. However, depending on the constraints of your curricular structure, students may still benefit from this session at other times in their education (for example, during their core clerkships, as they encounter this issue in real time).

This decision will guide how you modify the session and/or pre-work materials such that they are developmentally appropriate for learners. For example, first-year medical students may have very little context for the content of notes or potential pitfalls when writing them. This group of students will likely require more preparation materials. Clinical students, by contrast, have mastered some of these skills and may have seen firsthand the harms of non-patient-centered documentation, and thus may benefit from discussion that draws upon their personal experiences.

**>3 months prior to the session: Recruit facilitators and develop faculty development guide**

Recruit faculty facilitators, ideally those who are interested in medical education and patient-centered documentation. It is especially helpful if faculty have worked at an institution with a longer history of medical record sharing with patients. Additionally, recruit student facilitators who have completed their core clerkships and therefore can speak to their experiences with patient-centered documentation. Near peer mentors can be particularly helpful for encouraging other students to engage in discussion, as their experiences often feel most relevant to younger students. Residents and fellows can also be recruited, as they can provide valuable insight into the process of working with medical students and receiving upward feedback themselves. At our institution, our faculty recruited students and residents with whom they’d met in clinical and classroom settings and who’d expressed interest in patient-centered documentation.

**1-3 months prior to session: Develop relevant materials**

- **Session materials:** Consider incorporating real (de-identified) examples of notes/clinical scenarios that emerged for faculty or student facilitators. Update the facilitator guide to fit your specific institutional context. Determine if and how much pre-work to send to your learners based on your time constraints and their existing level of knowledge about OpenNotes.
- **Assessment and reflection materials**: Determine whether you would like to evaluate your learners and/or assess the success of your program, such as by administering pre- and post-session surveys. Additionally, you may consider longer-term follow up, such as meeting with students at a later point in their medical education so that they can reflect on the impact of the programming.
- **Discussion group questions**: Amend discussion questions to fit your learners’ educational stage. Consider suggested questions from student or faculty facilitators.

**1 week prior to session: Facilitator development**

Meet with student and faculty facilitators to discuss the content for the session. Ensure that there is time for questions and discussion that might inform the final session agenda.
