# Supplementary material for: A Patient-Centered Documentation Skills Curriculum for Preclerkship Medical Students in an Open Notes Era
Source: MedEdPORTAL. 2024 Mar 26;20:11392. doi: 10.15766/mep_2374-8265.11392 (PMC10963659; doi:10.15766/mep_2374-8265.11392)
Supplement: Supplementary file 1 — Checklist of Best Practices.docxRubric.docxFacilitator Guide.docxCourse Planner Implementation Guide.docxAsynchronous Module folderStudent Guide.docxWritten Documentation Guide.docxStudent Session Slides.pptxSample Note.docxModel Note.docxAttitudinal Survey Questions.docxKnowledge Questions.docx [file mep_2374-8265.11392-s001.zip › E. Asynchronous Module/content/index.html]

Open Notes and the Importance of Medical Record Transparency
