# Supplementary material for: A Patient-Centered Documentation Skills Curriculum for Preclerkship Medical Students in an Open Notes Era
Source: MedEdPORTAL. 2024 Mar 26;20:11392. doi: 10.15766/mep_2374-8265.11392 (PMC10963659; doi:10.15766/mep_2374-8265.11392)
Supplement: Supplementary file 1 — Checklist of Best Practices.docxRubric.docxFacilitator Guide.docxCourse Planner Implementation Guide.docxAsynchronous Module folderStudent Guide.docxWritten Documentation Guide.docxStudent Session Slides.pptxSample Note.docxModel Note.docxAttitudinal Survey Questions.docxKnowledge Questions.docx [file mep_2374-8265.11392-s001.zip › F. Student Guide.docx]

*Appendix F: Student Guide*

**A Patient-Centered Documentation Skills Curriculum for Pre-Clerkship Medical Students in an Open Notes Era**

**Session Learning Objectives:**

1. Define the 21^st^ Century Cures Act and the research of open notes and patient engagement.
2. Identify language used in discussing patients and documenting patient concerns that could be harmful to patients, create unwanted bias, or adversely affect other health professionals’ understanding of patients.
3. Demonstrate a patient-centered approach to documentation and increase self-reported preparedness in using nondiscriminatory, non-judgmental, and inclusive language in clinical notes.

Students should review the below three items prior to the session to fully engage in the material being taught and to understand the format of the two-hour workshop.

**Pre-Session Preparation & Review:**

1. **Student Guide** (Appendix F)
2. **Asynchronous module** (Appendix E): https://rise.articulate.com/share/qnCYgOHtjWJ3FJmls5RBQXcc_OAk_Hm7#/
3. **Written Documentation Guide** (Appendix G)

**In-Session Agenda:**

| **Estimated duration** | **Session Component** |
| --- | --- |
| **50 minutes** | **Part 1: Large Group Session** |
| - 20 minutes | Overview of Clinical Notes |
| - 30 minutes | Discussion: Takeaways from Asynchronous Session on Open Notes |
| **10 minutes** | **Break and transition into small groups** |
| **60 minutes** | **Part 2: Small Group Session** |
| - 35 minutes | Introduction & Dissecting Prepared Note |
| - 25 minutes | Best Practices Checklist & Rewrite Note Using Rubric |

**In-Session Materials:**

1. Student Session Slides for Facilitator use (Appendix H)
2. Checklist of Best Practices (Appendix A)
3. Rubric (Appendix B)
4. Sample Note (Appendix I)

**Post-Session Handouts:**

1. Student Session Slides for reference (Appendix H)
2. Model Note (Appendix J)
