# Supplementary material for: A Patient-Centered Documentation Skills Curriculum for Preclerkship Medical Students in an Open Notes Era
Source: MedEdPORTAL. 2024 Mar 26;20:11392. doi: 10.15766/mep_2374-8265.11392 (PMC10963659; doi:10.15766/mep_2374-8265.11392)
Supplement: Supplementary file 1 — Checklist of Best Practices.docxRubric.docxFacilitator Guide.docxCourse Planner Implementation Guide.docxAsynchronous Module folderStudent Guide.docxWritten Documentation Guide.docxStudent Session Slides.pptxSample Note.docxModel Note.docxAttitudinal Survey Questions.docxKnowledge Questions.docx [file mep_2374-8265.11392-s001.zip › G. Written Documentation Guide.docx]

*Appendix G: Documentation Guide*

written documentation guide

*Developed at Harvard Medical School 2022*

**Table of Contents**

Introduction to the Written Note………………………………………………… pages 1

Structure & Components of the Note…………………………………………. pages 2-10

Patient-Centered Documentation Practices………………………………… pages 11-14

Special Circumstances…………………………………………………………………. page 15

Checklist of Best Practices…………………………………………………………… page 16

Grading Rubric …………………………………………………………………………… page 17

Sample Written Note…………………………………………………………………… pages 18-21

**Introduction to the Written Note**

One of the foundational clinical skills necessary for the care of patients is the written note. The written note serves as a record of the encounter with the patient, a platform in which to express your thoughts and recommendations, and a mechanism to share information with the patient and other healthcare professionals involved in the patient’s care.

Since April 2021, new federal rules mandate that all patients (with several permitted exceptions) are offered rapid, online access to their clinical records, including the notes written by clinicians (‘open notes’). Extensive survey research demonstrates that patients who review their records and read their notes feel more involved in and knowledgeable about their care, better prepared for visits, and report being more likely to follow their clinicians’ advice.

There are many types of clinical notes depending on the encounter between the patient and the healthcare professional. Some examples include office notes, hospital admission notes, hospital progress notes, telephone notes, telemedicine visit notes, and emergency room visit notes. During preclinical and clinical years, medical students will meet patients in a variety of settings, including outpatient offices (for example, in the context of a primary care clerkship) and inpatient hospital settings (for example, during courses introducing the student to basic clinical skills or a surgery or medicine clerkship). Written notes are commonly taught with an initial focus on the history and physical note (also known as the “H&P” note). These notes often capture interactions with patients who are being seen for an initial visit, follow-up visit, or urgent care visit in the office setting, and with patients who are hospitalized for a specific reason. In courses designed to introduce basic clinical skills, students commonly learn all the components of the H&P: written history, physical exam, summary statement, assessment, and plan.

This guide aims to provide students and faculty with a comprehensive overview and approach to best practices with written documentation. This includes both organizational considerations in the structure of the note as well as a patient-centered approach with language and tone. As students will learn in their introductory clinical courses, the written note aligns with oral presentations: both structurally and in having a patient-centered focus.

The written patient note and the oral presentation are not one and the same. This is particularly evident when presenting the history beyond the history of present illness (HPI). As described by Dr. Clifford Packer in *Presenting Your Case: A Concise Guide for Medical Students*, the written patient note is a:

*“meticulous and detailed written report of the patient’s history, complete physical exam, allergies, medications, family history, social history, and review of systems … which serves as an important archive of baseline information on the new patient.”*

In contrast, the oral presentation is a:

*“focused and carefully edited production that both gives the essential clinical information and builds the argument for a particular diagnosis. It includes a concise and focused summary of the patient’s history …. It is not meant to be all-inclusive and archival.”*

**STRUCTURE & COMPONENTS OF THE NOTE**

The typical patient encounter note in the office and hospital setting is structured in a specific way, broken down into a flow which systematically builds towards the patient’s possible diagnosis and recommendations for next steps in diagnosis and plan. The note begins with a description of what is bringing the patient to medical attention, followed by a further description of their symptomatology and other background medical information (as applicable). After the history section, an objective assessment of the patient is noted – the physical exam and relevant laboratory and test results. The note ends with a summary, assessment, and plan of the patient’s presenting concern, which addresses all pertinent findings on the physical exam and other data, and initial diagnostic considerations.

Not all components of the written note will be included in every clinical note, depending on the situation. Specifically, the full written history with all its components is typically reserved for new patient visits to the hospital and/or office. Similarly, a full head-to-toe physical exam description is also reserved more for the initial encounter or primary assessments. Follow-up notes ('progress’ notes) and urgent care notes typically focus on an abbreviated and pertinent written history (in the same order as noted below) and focused physical exam.

1. **Chief Concern**
   1. ***What this is:***
      1. 1-2 main symptoms that have brought the patient to medical attention (i.e., reason for coming to the doctor’s office today, or reason for hospital presentation, or main concern they have)
   2. ***Recommendations:***
      1. *Use the patient’s words* – this is usually the patient’s main presenting symptom(s) or concern in their words. Sometimes this only comes out at the end of your interview. It is not necessarily the first statement made by the patient.
      2. *Start from the beginning* - When someone presents with chest pain, make the chief concern “chest pain” as opposed to “MI” or “PE.” Even if you know the diagnosis at the time of writing the note, you have to build a case for why you think the patient has a certain condition throughout the note.
      3. *Use the phrase “Reason for admission”* instead of “chief concern” when there is an objective reason the patient was admitted (such as “elective right-heart catheterization” or “abnormal labs”). These are usually not chief concerns of the patient.
2. **One-liner/opening sentence**
   1. ***What this is:***
      1. WHO the patient is; pertinent past history and risk factors; main symptoms bringing to medical attention (i.e., chief concern); temporal pattern (can be descriptive such as “progressive”, or numerical such as “1 week” – which is used and when will depend on the context and situation)
   2. ***Recommendations:***
      1. *Humanize your patient* - Consider writing “Ms. Sanders is a 94 woman with stage III lung cancer who presents with…” rather than “94F with stage III lung cancer” because it humanizes the patient to use her name.
         1. For write-ups that are being uploaded and sent to non-HIPAA platforms (Canvas, email), please use patient initials ONLY and/or ensure no identifying information is included in the note.
      2. Always use the patient’s self-identified pronouns. Many electronic records list this based on patient report.
      3. Consider thoughtfully whether gender needs to be in the one-liner given the limitations of full expression of non-binary genders experienced by individuals.
      4. We recommend not including race in an opening sentence or one liner.
      5. You can also add elements of social history here to humanize the patient. “M.S. is a 94-year-old former grade school teacher, with stage III lung cancer…”
      6. *The opening line should only contain what matters most for your argument* - Only include the pertinent past medical history (PMH) (not every condition the patient has) in the one-liner. “Ms. Sanders is a 94W with stage III lung cancer and recent diagnosis of PE who presents with hemoptysis” is much more relevant than “Ms. Sanders is a 94W with stage III lung cancer, hypothyroidism, hyperlipidemia, colon polyps, ankle fracture s/p ORIF (1984) and recent diagnosis of PE who presents with hemoptysis.”
3. **History of Present Illness (HPI)**
   1. ***What this is:***
      1. It tells the story of the patient’s chief concern. It weaves the information provided by the patient into a coherent narrative that includes a chronological description of the manifestations of illness, attempted interventions, and patient interpretations. The HPI narrative requires you step back and organize all information into a coherent story. It is not a transcript of what the patient says.
   2. ***Recommendations:***
      1. Give the *narrative*, with all the detail possible, creating a picture/movie in the minds of your audience as to what events transpired.
      2. *Be clear* where you got the history – from the chart review, from the patient, from the family, report of the nursing home you called, etc.
      3. Always *double check* any chart obtained information with the patient – it may be wrong!
      4. Keep things in *chronological order*, which means telling pertinent past history (such as a recent hospital admission) before getting to the HPI. In some instances, it may make more sense to reverse the order, such as when providing information on a chronic condition diagnosed many years ago.
      5. Don’t say “Last Wednesday” or “yesterday.” Use dates or reference of number of days prior to admission instead for clarity.
      6. What about review of symptoms (ROS) in HPI? Whatever organ systems are important for your HPI should be listed with complete ROS here. Everything else on ROS should go in the later section. For example, a patient with shortness of breath should have ROS for general, pulmonary, cardiovascular (CV), and hematology listed in HPI, as well as additional organ systems depending on the presentation.
      7. Include pertinent information from other sections of the history (PMH, allergies, meds, social history (SH), family history (FH)) into the HPI *if* it is relevant to the presenting concern. Otherwise, this information should stay in its specific sections.
      8. For all patient concerns, make sure you write a paragraph that includes all the key information to answer the following questions. A helpful starting point can be the mnemonic “OPQRSTAA.”
         1. How did the concern start?
         2. When did it start?
         3. Where did they feel the pain start?
         4. Does anything seem to make it better?
         5. Does anything seem to make it worse?
         6. How often does it occur?
         7. How long does it last when it does occur?
         8. If it always is present – ask if the intensity varies, if so – how much?
         9. Intensity: Can they articulate on a scale? Can they describe how much it distracts them during their day?
         10. Did it seem to be associated or correlated with anything common in their day – eating, hunger, breathing, urinating, stooling, menstrual periods, sleeping, exercising, etc.
         11. If patients do not recall specific details, ask them to recall one specific instance and to walk you through what they remember of that one specific experience.
         12. Did they try any medication, over the counter item, vitamin, herbal remedy, or other practice to try to make it better? If so, what was the effect?
         13. Have they ever had anything like this before? If so, when? What happened at that time?
         14. Have they seen any medical providers for this concern? If so, what do they recall of those evaluations?
         15. What do they think it is? What are they worried it may be?
4. **Additional information into the HPI**
   1. ***Emergency department (ED) course***
      1. Patients may spend time in the ER prior to coming to the floor, thus the events in the ER are part of the story of their care. This will typically be added in a designated section below the main HPI.
      2. The important information to get across about an ED course are the patient’s *clinical trajectory* (HR improved from 120s to 90s with 2 liters of normal saline IV fluids) and the *diagnostic and therapeutic decisions* made (“Chest x-ray revealed a possible airspace opacity, so patient received IV antibiotics” or “Patient was hypoxic and short of breath; 40mg IV Lasix given with urine output of 2 liters over two hours with subsequent resolution of the hypoxemia”).
      3. Above all, *stick to facts* rather than paraphrasing the ED team’s interpretations of data. Say, “They gave one dose of levofloxacin 750 mg” rather than, “they thought it was pneumonia on the chest x-ray, so they gave her levofloxacin.” We can never fully know the thinking of other clinicians caring for our patients. It is ideal to stick to objective facts in your reporting of data.
      4. When you add documentation of what the patient’s vitals were upon arrival to the floor, consider adding the time course for clarity (ER door to floor time approximation).
   2. ***Outside hospital data***
      1. Any outside hospital data may need to be covered as a paragraph in your HPI if relevant.
      2. You should note how you obtained the data, and list the fact you collected, without adding commentary (ex – “patient was given 3 days of ceftriaxone as documented in the outside hospital records”)
      3. Do not write statements that could suggest judgment or your opinion, “*They thought she had pneumonia, so they gave her ceftriaxone for three days, but she never got better and then they did a CT chest and sent her here because it showed they missed a lung cancer.”*
5. **Past medical and surgical history**
   1. ***What this is:***
      1. Encompasses significant illnesses (childhood & adult), operations, reproductive history, other hospitalizations, serious injuries; with dates/duration/therapies noted as applicable.
   2. ***Recommendations:***
      1. Avoid copying and pasting automated problem lists from the electronic medical record (EMR). Verify all information with the patient!
      2. If objective parameters for a medical problem are readily available, list those next to the condition (such as last hemoglobin A1c for diabetes)
      3. It’s okay to blend medical and surgical history together sometimes. It makes more sense to write “critical aortic stenosis status post mechanical AVR in 2005” than to list critical AS and aortic valve replacement as separate conditions.
      4. It’s also okay to put medications in the problem list to convey a sense of severity – ex. DM2 controlled on insulin (vs diet); HTN usually controlled per PCP notes on 4 drug regimen, goal <130/80.
6. **Medications**
   1. ***What this is:***
      1. A complete list of medications (prescribed, over the counter, supplements) the patient is taking when out of the hospital – with doses, duration (if a limited course, such as antibiotics)
   2. ***Recommendations:***
      1. Again, do not copy and paste automated med lists from the EMR. This can turn into a mess!
      2. Confirm with patients, their family, nursing home, or confirm refill history with pharmacists via phone calls.
      3. Use *generic* medication names. Always list dose and frequency. It’s also okay to annotate if the patient is actually taking it or not.
      4. Consider *grouping* medications by indication to both show your understanding of why the patient is prescribed the meds and to enhance your remembering their medication list (e.g., cardiovascular: aspirin 81 milligrams daily, simvastatin 40 milligrams daily; psychiatric: sertraline 50 milligrams daily; lorazepam 1 milligram HS prn insomnia).
      5. Common abbreviations regarding dosing intervals include: QD (daily); BID (twice daily); TID (three times daily); QID (four times daily); Q”x” hours (every “x” hours); PRN (as needed). It is **strongly recommended** you write out the full word (“daily”, “twice daily” etc.) to get into the habit of not using abbreviations in your note.
7. **Allergies/adverse reactions**
   1. ***What this is:***
      1. Documentation of the patient’s known allergies/adverse reactions to medications, substances, foods.
   2. ***Recommendations:***
      1. Do your best to determine what the reaction to the medication was. In many cases, the type and severity of drug reaction will determine if a patient can receive that med (or similar ones) again.
      2. Double check with the patient or their family what is known or unclear.
      3. It is ideal to list intolerances as well when they may be pertinent.
8. **Family history**
   1. ***What this is:***
      1. Information about the patient’s first-degree relatives:
         1. If living: age and current state of health.
         2. If dead: age at death and cause of death.
         3. Significant history of health problems
      2. Information about more distant relatives with potentially heritable medical or psychiatric problems.
      3. An indication of adoptions (either the patient’s or other family members’) or other biological and social relationships, such as step-relatives.
   2. ***Recommendations:***
      1. Writing “non-contributory” or “not relevant” is not acceptable in a note.
      2. Make sure you ask about diseases relevant to the patient issue; this info supports your argument.
         1. Ex. Patient with cardiovascular chief concern, make sure you ask them about family history of cardiovascular issues.
9. **Social History**
   1. ***What this is:***
      1. Description of your patient’s meaningful relationships and social supports, occupation, religious or spiritual beliefs and practices, financial issues, immigration history, occupational exposures, substance use history (tobacco, alcohol, recreational substances), and other details that help to define the patient in relation to society at large. It should also reflect cultural context and issues.
   2. ***Recommendations:***
      1. As a student, you may have the time to know the patient as a person better than anyone else on the team. This is the section into which you can place some of those personal details.
      2. The social history is often a key consideration when planning for discharge and care of the patient after this hospitalization.
         1. Ex. Ms. Jones lives alone, ambulates with a walker around her home, has 3 cats and a supportive neighbor, and receives meals on wheels.
         2. Ex. Mr. Smith lives alone, is independent of ADLS and iADLS, jogs three times a week, and has 2 children who live downstairs and see him daily.
10. **Review of systems**
    1. ***What is it:***
       1. A comprehensive list of closed-ended questions organized by system. There are 14 recognized systems for documentation of ROS:
          - Constitutional symptoms (i.e., fever, weight loss, vital signs)
          - Eyes
          - Ears, nose, mouth, throat
          - Cardiovascular
          - Respiratory
          - Gastrointestinal
          - Genitourinary
          - Musculoskeletal
          - Integumentary
          - Neurological
          - Psychiatric
          - Endocrine
          - Hematologic/Lymphatic
          - Allergic/Immunologic

- 1. ***Recommendations:***
     1. The final ROS should be all body systems that are not pertinent to chief concern, or primary reason for hospitalization. This is where you list out anything else that you do not think is critical or relevant to the patient’s primary needs during hospitalization.
     2. List out all the questions you asked. The phrase “10-point ROS reviewed in detail and negative except as noted in HPI” does not belong in a student note!
     3. List out the systems you that you review with the patient. Any pertinent positive or negative symptoms related to the chief complaint body systems belong in the HPI, but other organ system symptoms should be listed in this section. This is a way of documenting your argument about what you think is going on. If you feel the chief complaint of shortness of breath could involve a cardiac, pulmonary, hematologic, or other system, then all the ROS questions for those body systems should be noted in your HPI as either present or absent. Any system that you feel is not likely to be related to the chief complaint, should have a complete ROS listed but you place it in the paragraph later in your documentation, separate from the HPI.
        1. Example for person with cc: shortness of breath (SOB)
           1. ROS:

General: see HPI

CV: see HPI

Pulm: see HPI

GI: no diarrhea, no constipation, no nausea or vomiting or change in appetite

Derm: no rashes or new skin lesions

Neuro: no headaches, weakness, or vision changes

MSK: no joint pain or swelling

1. **Physical Exam**
   1. ***What this is:***
      1. Objective and systematic documentation of your findings from performing a full or focused physical exam on the patient.
   2. ***Recommendations:***
      1. The physical exam that you perform and document should be *tailored to the patient’s chief concern*.
      2. You should document the foundational physical exam, as well as the comprehensive exam for the organ systems pertinent to the chief concern.
         1. Ex. For a person with shortness of breath, include the foundational exam plus all comprehensive exam components for cardiovascular and pulmonary.
      3. *Create a picture of the patient* - For someone presenting with shortness of breath, commenting in the “general section” that the patient is “not using accessory respiratory muscles; conversing comfortably, able to speak in full sentences” is extremely useful. Also helpful would be: The patient was “leaning forward in tri-pod position, coughing during interview, pursed lip breathing, speaking in short phrases only.”
      4. *Be Objective!* Avoid saying that a component of the physical exam is “benign” or “non-focal.” This is an interpretation, not an objective statement of what you found one exam. Similarly, do not write that a murmur is an “ejection” or “flow” murmur; these terms are interpretations and mean different things to different people. Stick to describing what you hear (ex. Grade 3/6 early systolic murmur).
2. **Data**
   1. ***What this is:***
      1. Labs, imaging, microbiology, electrocardiograms (ECGs), pathology/cytology, any other reports of tests performed.
   2. ***Recommendations:***
      1. Be thorough and complete. Ensure your documentation matches what is listed in the actual record.
      2. Many EMRs will automatically import the data based on what you tell it to import. If this is the case, aim to import only new data that has not yet been discussed/reviewed and/or old data that is significant to the patient’s care.
3. **Assessment**
   1. ***What this is:***
      1. A summative statement that argues what you think the diagnosis is and how you explain: “Why this patient, why now?”
   2. ***Recommendations:***
      1. The assessment should NOT be the same sentence as the opening statement of your HPI
      2. We recommend following the framework below:
         1. [Name] is an [age] [gender] w/ history of [relevant PMH/risk factors] presenting with [temporal pattern] [cc] and [key HPI items] found to have [key data].
         2. Example statement: *Ms. MT is a 35-year-old woman with no significant past medical history who presents with 1 day of severe L sided chest pain, shortness of breath, and a racing heart that developed after a long flight and is found on physical exam to have tachycardia, hypoxia, L lower leg swelling, redness, and skin tightening from the ankle to the knee.*
4. **Problem List**
   1. ***What this is:***
      1. It is the identification, grouping, and labeling of the patient’s medical concerns.
      2. The problem list should include any “problem” that you uncover in your time with a patient. This may include symptoms noted by a patient as part of the HPI, concerns that arise in the patient’s past medical/surgical history, medications, allergies, family history, social history, or review of systems, any abnormal physical exam findings, or any abnormal or concerning lab or imaging results.
   2. ***Recommendations:***
      1. Have one! List everything that is a concern of the patient on history, exam, labs, imaging, etc.
      2. If you omit a problem list, you are more likely to miss puzzle pieces important for the patient diagnosis and care.
      3. Organizing the info may lead to a long list – but it helps you see pathophysiologic connections and what could be grouped toward supporting one diagnosis and refuting another. *“Lumping vs. splitting”* in the problem list is something you can refine over time as you learn more about pathophysiology of diseases. As an early learner, we encourage you to split the problems in the list so as not to prematurely group items together; as your clinical experience and knowledge grows, you will begin to lump and prioritize problems together based on your understanding of how they relate to each other.
      4. Example problem list based on patient MT above (all problems “*split*”):
         - L sided chest pain after a long flight, Shortness of breath
         - Racing heart
         - Contrast allergy
         - Family history of breast and ovarian cancer
         - Prior history of trauma
         - Tachycardia
         - Tachypnea
         - Hypoxia
         - Swollen L leg
         - Redness and tightening of the skin of the L leg
      5. Example problem list based on patient MT above (all problems “*prioritized and lumped*”):
         - L sided chest pain after a long flight, Shortness of breath, Racing heart, Tachycardia, Tachypnea, Hypoxia, Swollen L leg, Redness and tightening of the skin of the L leg
         - Contrast allergy
         - Family history of breast and ovarian cancer
         - Prior history of trauma
5. **Plan**
   1. ***What this is:***
      1. This is your recommendations for each problem identified for the patient, organized by differentials being considered for this problem/symptom (if applicable); what diagnostic next steps you recommend being performed to help further elucidate the diagnosis (if applicable); and your recommended plan for both symptom management and diagnosis treatment.
   2. ***Recommendations:***
      1. Organize by problem (in order of decreasing severity/importance), listing pathophysiologic connections, differential diagnosis, diagnostic plans, and therapeutic interventions for each problem in the list.
      2. Should include name of problem (symptom, sign, or diagnosis when known), likely etiology (differential diagnosis discussion), diagnostic plan, therapeutic plan.
         1. Example for patient MT’s # 1 problem:
            1. Chest pain:

Differential includes pulmonary embolism, acute myocardial infarction, pneumonia, and costochondritis. Given the recent long flight, tachycardia, hypoxemia, and lower extremity symptoms, a pulmonary embolism is highly likely to be the etiology of her chest pain. Given no risk factors for coronary artery disease, no family history of CAD, lack of ischemic changes on EKG, it is less likely this is an acute myocardial infarction. Given no fever, cough, or infiltrate on chest x-ray, pneumonia is unlikely……and so forth.

Diagnostics: Plan for a CTA to assess for a pulmonary embolism and a LENI to assess for DVT; will also repeat an EKG and check cardiac biomarkers to rule out an AMI.

Therapeutics: Will plan to start anticoagulation pending CTA results, for now will keep on telemetry and continuous O2 saturation monitoring.

- - - - 1. For a chronic problem, the etiology is sometimes less important, but a therapeutic plan and reasoning should always be included.

Example chronic problem:

Hypertension, essential: Goal BP 120s/70s, will hold home Lisinopril given current AKI and monitor. Will continue to reassess.

**Patient-Centered Documentation**

In the era of open notes, it is vital to be cognizant of how we represent our patients and the clinical encounter in written and oral form. Although open notes address an important ethical issue - ensuring that patients have access to what is written by clinicians in their record - they also present novel ethical and practice dilemmas. Words matter, and inclusion of social determinants of health, medical vernacular (e.g., “patient denies”) and medical terms (e.g., “obesity”) are sources of concern, with some persons potentially at greater risk of feeling offended. The clinician must balance the risks of harming the relationship with the risk of undermining clinical communication and clinician autonomy. Accompanied by important repercussions for trust in providers, stigmatizing language, or insensitive notes, may also especially affect persons from sexual/gender, cultural, ethnic, or religious minorities. Other dilemmas arise in complex family dynamics, where some individuals may expect to access their family member’s notes. Studies show that many clinicians report being less candid in documentation after the implementation of open notes, and about a quarter of physicians change how they write differential diagnoses. Upholding accuracy in clinical record-keeping remains a clear imperative.

The below is a summary of general principles to approaching documentation in a patient-centered manner. These principles are well documented in literature and are also directly reflective of feedback from patient advocates along with physician experts in open notes.

General Principles for Effective Practices:

1. **Curiosity**. Come from a place of curiosity when interacting with patients - first and foremost. Notes are not a transcription of a visit. Document the key information. Be a detective. Investigate.
2. **Set expectations.** Let the patient know from the start that you will be asking questions to understand how the patient wants to be represented in how we talk and write about them. “You will be able to read your notes after they are signed. We are all still learning how to write notes. Our goal is to try our best to write what is important for all the people caring for you and managing your health to know what is important for this time in your care.” “I want to do my best to write about you and speak about you in ways that capture the important medical information, while understanding you as a person. I want you to feel seen and heard in all that we do.”
3. **Be honest.** Write what you discuss. Discuss what you’ll write. Don’t put information in a note that you never mentioned to the patient. Balance your documentation of clinical reasoning vs. what you discussed with the patient.
4. **Write from your perspective**. Your note is your argument about what is important to know for care now – the documentation is your opinion, to the best of your ability. This is what we all strive for; our specialist colleagues do this well. It should not be viewed as the final or full truth about a person. You can write your clinical reasoning as “I think or we think (when including your attending) ….” And represent your thoughts and reasoning without invalidating the patient’s perspectives or understanding. “My (our) recommendations at this time are….”
5. **Avoid excessive use of abbreviations.** A statement such as “52M with HFrEF s/p biV ICD, PAF (on a/c), ESRD c/b hyperK…” ends up looking like alphabet soup. When in doubt, write it out! Use only officially accepted medical abbreviations, regardless of what you see others do.
6. **Be accurate**. Ensure the note represents up to date and accurate information. Avoid the temptation of cut & paste from the chart; especially without verifying information with the patient. **Check your spelling.** The words “guaiac,” “ophthalmology,” and “arrhythmia” have gotten the best of many of us.

Guiding Principles by Section

**Chief Concern (CC) and History of Present Illness (HPI)**

1. Ask the patient how they want to be identified and refer to patients as they identify themselves.
   1. Honorifics and gender identity should never be presumed. Always ask patients how they identify and how they want to be identified in the record. Do not refer to someone as Mr. or Ms. or male or female unless you have confirmed how they identify. This is most easily done when you are first introducing yourself to a patient.
   2. Patients may want to be identified by their important life roles.
      1. Ex. 1: Ms. Jones is a 23 y/o female presenting to the clinic with concern for….
      2. Ex 2: Mr. Samson is a 58 y/o teacher and father, presenting with chest pain….
2. Use “person first” language
   1. People have diseases or disorders; they are not labeled as diseases or disorders. The person is separate from the symptoms and the disease.
      1. Ex. 1: Mr. Smith is a 42 y/o man who has diabetes, hypertension, and coronary artery disease – (not - Mr. Smith is a 42 y/o diabetic with hypertension, and coronary artery disease)
      2. Ex. 2: Ms. Johnson is a 35 y/o woman with paraplegia after a spinal cord injury in 2010 – (not Ms. Johnson is a 35 y/o paraplegic)
      3. Ex. 3: Sam is 30 y/o teacher with prior experience of substance use disorder and depression, presenting with change in mental status…
3. Introductory HPI sentences should mention the factors important to understanding and planning the care of that person at that point in time. These are dynamic and change over time. Be thoughtful and deliberate about including demographic factors, epidemiologic factors, medical and social history in these key sentences.
4. Recognize that commonly used phrases connote bias, contribute to judgments, and propagate misunderstandings of diseases.
   1. Chief concern (not complaint – patients are not complainers)
   2. Patient reports or tells us (not patient claims, patient endorses). Better phrasing is “patient does not report x, y, z” or “patient tells us she has never had X, Y, Z” (not patient denies)
   3. Many terms are misinterpreted and can represent or propagate bias. Understand the words you are using and the possible interpretations in medical and common vernacular. Avoid the terms compliance, adherence, AMA (against medical advice), and acronyms (SOB, FOB).
   4. Be cautious about using quotation marks in the note, even if to capture exactly what the patient said. This can be interpreted negatively by the patient and others reading the note. When considering patient use of particular words, it is ideal to use that as a time to clarify meaning with the patient, rephrase and share your rephrasing to check your understanding with them. Summarizing information back to the patient always helps us ensure we get it right. “I want to make sure I understand what you mean by X and write this down so others reading your notes understand it as well.” “Let me summarize what I heard as I write it down/type it and let me know if you want me to correct anything.”
   5. Avoid what could be considered coded language – poor historian, low health literacy, judgements about explanatory models, “patient is quite involved in their care” “patient has high health literacy.” Better phrases would be “(patient name) appreciates understanding our thoughts and partnering in their care.” “Patient is not able to recall the events that I asked about today. Her daughter assisted with the interview and tells me….”

**Past Medical History (PMH)/Medications/Allergies/Family History (FH)**

1. Write what you confirmed with the patient. If information is from the chart, state that it is from chart. Do not assume chart history is correct. Always confirm with the patient and/or family.

**Social History (SH)**

1. Always discuss what is important to write regarding social history. Not all social history is important to write in the chart note. Social history changes over time. Check and update past records of social history in the EMR. Be cautious about documenting subjects that require much skill and reflection.

**Review of Systems (ROS)**

1. If the symptom reported has a possible physiologic connection to the chief concern, include it in the HPI section. Note: it is common that something reported on review of systems is significant and requires its own paragraph of a second HPI. If a symptom seems minor and/or completely unrelated to the chief concerns, it may be listed in the separate ROS section as incidental information.

**Physical Exam**

1. Documenting the physical exam findings should focus on objective information that informs clinical reasoning.
   1. Be cautious about documenting details on appearance as this can be highly subjective. Document only what informs clinical reasoning – not judgments that are subjective.
      1. Ex. 1. “Patient is disheveled appearing with uncombed hair and coffee stains on front of shirt with strong stench of body odor.” While some people may argue this conveys your “objective” description of the patient, it can convey bias. All sensory observations are subjective. It also does not inform clinical reasoning or planning for care of the patient. If the appearance of the patient raises concern for you, we propose that is a signal for further curiosity and inquiry with the patient. Perhaps the patient will share information that helps you identify barriers to self-care, or access to resources. These are important factors and better described in the HPI or social history – rather than in the exam.
      2. Ex 2. “Patient is seen today sitting up in bed, reading newspaper, with breakfast plate empty, tray at bedside. Speaking clearly in full sentences without conversational dyspnea.” This helps inform the picture of the patient at that point in time.
      3. Ex. 3: “Patient is a pleasant woman in no acute distress.” The word ‘pleasant’ is a judgment and has no bearing on your clinical reasoning of the patient. Though the patient may be in fact, pleasant, this can imply other patients are NOT pleasant.
      4. Describing a patient’s body habitus may be best to do solely with objective data (weight, BMI, height) rather than words that can be stigmatizing (“obese”, “morbidly obese”)

**Data**

1. All data obtained should be explained to the patient in a timely manner after results are available. It is not ideal for patients to learn about test results or reports after reading your notes and/or reviewing in the record without context.

**Assessments/Summary Statements**

1. Assessment statements, summary statements should mention the factors important to understanding and planning the care of that person at that point in time. These are dynamic and change over time. Be thoughtful and deliberate about including demographic factors, epidemiologic factors, medical, and social history in these key sections.
2. Labeling diagnoses is important for conveying understanding of disease processes, management, and reimbursement for care. When recording a diagnostic label, mention to the patient the words, meaning, and your reason for using it. “CHF, CKD, AKI.” Remind patients they can ask questions about labels and that no label should be a surprise. Encourage them to share their questions after reading the note at the next visit or another day.
3. Be sure to present your reasoning for diagnoses, differential diagnoses, what you are considering for diagnoses and share them with the patient. Do not list a possible diagnosis in a chart note without mentioning it to the patient. Write what you discussed.

**Plans**

1. Diagnostic and therapeutic plans should be clear. Action items the patient can understand, convey to others, or act upon themselves are always helpful for partnership.
2. Add empowering language where possible: “she has not been able to lose any weight yet.” “He has not reached remission of cancer yet.” Document shared decision making. Document any goal setting. Have some area of note / EMR that conveys action items that patient can understand, recall, use for preparation of next visit, or share with other care teams or loved ones.

**Consideration of Special Circumstances**

During introductory clinical courses and subsequent clinical training in clerkships and beyond, you will encounter patients for whom there are special circumstances that need to be taken thoughtfully into account when representing this information in the written note that is accessible to patients and their surrogate decision-makers (such as parents of an adolescent).

These scenarios are teachable moments for conversations with preceptors and patients, as for many of these circumstances, there is no right or wrong answer. Each healthcare professional may have their own approach and set of best practices. Students should always review these situations with their preceptors to ensure written communication is deliberate, thoughtful, and patient-centered.

The below list represents many of the areas we consider to be potentially sensitive. This is by no means an exhaustive list.

1. Social history: legal struggles, incarceration, concerns about marriage, children, or others. The note should include what is key for the patient’s care at that time.
2. Psychiatric history
3. Substance use history: reference years and state when in remission.
   1. For people who suffer from use disorders it is important to use “recovery language” – they can have recurrences (not relapses).
      1. Ex. She reports a recurrence of her substance use disorder. He has struggled to maintain remission.
   2. It is important to use person-first language – for example, we should write “48 y/o person with a history of injection drug use” (if applicable to the chief concern), rather than “48 y/o IV drug user”.
   3. When noting labs: a person can have a positive drug screen, or drug screen that showed concern for barbiturates (don’t say a “dirty” urine vs “clean” urine; describe all screens with caution in case of false positives)
   4. She reports she is drug free (or free from use of illicit and non-prescribed medications) at this time (not – she denies using recently).
   5. She was treated in 2019 with medically supervised withdrawal (not “she detoxed”).
4. Sexual history
5. Trauma history, intimate partner violence or abuse history
   1. Be mindful that individuals may choose to disclose a remote history of abuse to a specific clinician they trust and not to others- it is important to have an open discussion with the patient about their comfort with the documentation of this event in their notes.
   2. It may be beneficial to document the patient’s history without the word “trauma”, as this itself may carry stigma.
   3. Be mindful to document modifications to the physical exam or other aspects of the clinical encounter that can improve a patient’s experience and comfort in subsequent clinical situations, and also avoid posttraumatic stress reactivation in future clinical encounters.
6. Diagnosis labels - Weight struggles and obesity labels
7. Goals of care /advance care planning conversations
8. Barriers to care conversations
9. Pediatric and adolescent populations
10. Who has access to the chart in the patient’s world – don’t document something the patient would not want their parent, partner, family to read

**CHECKLIST OF BEST PRACTICES**

1. **Use person-first language.**
2. **Refer to your patient as how they want to be identified.**
3. **Avoid abbreviations and acronyms, especially if not officially approved by the hospital.**
4. **Say what you write, write what you say.**
5. **Verify past history information before including in the note.**
6. **Avoid words which can confer bias and judgment.**
7. **Keep physical exam descriptions objective.**
8. **Empower your patients with encouraging words and clear next steps.**
9. **Pay close attention to sensitive topics, including but not limited to sexual history, trauma history, substance history, mental health history.**
10. **Write from your perspective**

**Patient-Centered Documentation Assessment Rubric**

|  | **1** | **2** | **3** | **n/a** |
| --- | --- | --- | --- | --- |
|  | **None/absent** | **some** | **complete** |  |
| **WRITTEN HISTORY** | | | | |
| **Detailed HPI**  Defined as including a completed description of the chief concern (s) such as location, quality, severity, duration, timing, radiation, factors that aggravate or alleviate symptoms |  |  |  |  |
| **Descriptive HPI**  Defined as use of semantic and descriptive vocabulary such as acute or chronic, sharp or dull, continuous or intermittent |  |  |  |  |
| **Chronologic HPI**  Defined as telling a clear story that flows logically |  |  |  |  |
| **Contextualized HPI**  Defined as identification and inclusion of key findings from past, family, and social history and relevant other symptoms that might otherwise belong in later portions of the comprehensive history |  |  |  |  |
| **Complete comprehensive history**  Defined as complete and verified past medical, surgical, social, and family history, and complete review of systems |  |  |  |  |
| **WRITTEN PHYSICAL EXAM FINDINGS** | | | | |
| **Complete Physical Examination**  Defined as including all elements of the foundational head-to-toe exam performed during the patient encounter |  |  |  |  |
| **Key Physical Exam Findings**  Defined as including the hypothesis-directed pertinent exam findings based on the presenting chief concern(s) |  |  |  |  |
| **PATIENT-CENTERED DOCUMENTATION** | | | | |
| **Patient-Centered Description**  Defined as referring to the patient as they want to be described, using person-first language and social history   - Honorifics, gender pronouns, individual attributes - Patient is an individual WITH the condition, not defined by the condition (i.e., avoid “patient is a diabetic” and instead use “patient has diabetes”) |  |  |  |  |
| **Patient-Centered Language**  Defined as avoiding acronyms and words or phrases that connote bias, contribute to judgments, and propagate misunderstandings of diseases   - Deliberate use of judgment-free verbs (“concern” instead of “complaint”; “reports” or “says” or “tells”, instead of “denies”, “endorses”, “claims”) - Avoidance of terms which can confer bias (“compliance”, “adherence”, “AMA discharge”) - Exclusion of acronyms (“SOB”, “iso”, “f/u”, “r/o”, “MOP”) - Thoughtful & judicious use of quotation marks - Avoidance of language which can be considered coded (“poor historian”, “low health literacy”, “patient is quite involved in their care”, any judgements about explanatory models) |  |  |  |  |

Optional Narrative Feedback:

**SAMPLE WRITTEN NOTE**

**Chief Concern:**

Mr. A is a 64-year-old man with a history of pulmonary embolism x2 and type 2 diabetes who presented with fatigue and weakness.

**History of Present Illness:**

Mr. A had his first pulmonary embolism in 1999. At the time he felt acutely short of breath and immediately sought medical attention. In 2000 he had his second PE. His physician recommended a lifetime regimen of warfarin, but he opted for daily aspirin and turmeric instead.

Four weeks ago, while working his office job as a software engineer, he suddenly began to feel exhausted and weak. He reports no shortness of breath and instead described the sensation as “coming down with the flu.” He reports having had a dry cough. These symptoms worsened over 3 days, at which point he could no longer walk to the bathroom and called for an ambulance.

He was admitted to Metrowest Hospital for 3 weeks and found to have a new large pulmonary embolism. Throughout his hospital course he received anticoagulation including TPA, but his symptoms never fully resolved. He was discharged to rehab on 3L/min nasal oxygen and is now readmitted to BWH due to his persistent symptoms. He will be evaluated by surgery for consideration of clot removal via surgery.

**Past Medical History:**

- Pulmonary embolism (2 in the past 20 years)
- Type 2 diabetes
- Enlarged prostate
- Lactose intolerance
- Gout

**Past Surgical History:**

- Inguinal hernia repair

**Medications:**

- Rivaroxaban (oral)
- Tamsulosin
- Atorvastatin
- Lasix (oral)
- 3L/min oxygen nasal cannula

**Allergies:**

- Latex

**Social History:**

Mr. A lives alone in Wayland. He is divorced and has one son. He conveys at times feeling lonely and isolated without a lot of social contact. He has rarely had visitors during his hospital stays besides sisters that live in the area. He practices the Jewish faith. He is a software engineer and enjoys coding. He reports being sedentary 14 hours or more of the day at a desk. He reports that his diet usually consists of fruits, veggies, meats, and salmon.

**Family History:**

Maternal: none reported

Paternal: atherosclerosis, Parkinson’s

Brother: Parkinson’s

**Review of Systems:**

- General
  - Positive for weakness as per HPI.
  - Negative for weight change, fevers, chills, night sweats, appetite change, sleep difficulty.
- HEENT
  - Negative for headaches, dizziness.
  - Deferred asking about vision changes, hearing changes, discharge, hoarseness, and mouth sores.
- Respiratory
  - As per HPI.
- Cardiovascular
  - As per HPI.
- Gastrointestinal
  - Negative for abdominal pain.
  - Deferred asking about nausea, vomiting, diarrhea.
- Genitourinary
  - Positive for urinary catheter.
  - Deferred asking about frequency, urgency, dysuria, hematuria.
- MSK
  - Positive for weakness.
  - Negative for stiffness or pain.
- Hematologic
  - Deferred asking about easy bruising, bleeding.
- Skin
  - Positive for erythematous scaly rashes on his face.
- Neurological
  - Positive for mild tremors for the past two decades.
  - Deferred asking about numbness, tingling.
- Endocrine
  - Deferred asking about polyphagia, polydipsia, heat/cold intolerance.
- Psychiatric
  - Positive for depressed mood and increased anxiety.

**Physical Exam:**

***Vital signs***

Vital signs were not recorded.

***General***No acute distress, sitting upright in a chair comfortably, speaking full sentences without difficulty.

***Head***Head is normocephalic and atraumatic.

***Eyes***

No conjunctival pallor, injection, or erythema. Pupils are equal round and reactive to light and accommodation bilaterally. Sclera anicteric. Extraocular movements intact with no nystagmus. Visual fields intact.

***Ears***

No visible lesions of the external ear or external auditory canal. Tympanic membranes without erythema or bulging bilaterally.

***Nose***

No discharge, normal nasal mucosa without erythema or visible lesions

***Oropharynx***

MMM, no visible oropharyngeal lesions

***Neck***

Thyroid nonpalpable, no palpable cervical, supraclavicular, submandibular, auricular, or occipital lymphadenopathy, no stridor, or other upper airways noise

***Respiratory***
Increased work of breathing, resonant to percussion equally bilaterally posteriorly, audible left lower lobe crackles, clear to auscultation bilaterally anteriorly

***Cardiovascular***

JVP non-elevated, regular rate and rhythm, normal S1 and S2 with no murmurs rubs or gallops, deferred palpating for RV heave or PMI

***Abdominal***

Normal contour, normoactive bowel sounds, no-distended, non-tender to light and deep palpation in all 4 quadrants, did not assess for hepatosplenomegaly

***Skin***

Warm and dry, scaly erythematous rashes on face

***Extremities***

Could not feel dorsalis pedis or posterior tibial pulses, pitting edema, could not do proper exam due to compression socks

Deferred remaining exams including MSK and neurologic as the patient was anxious for surgery tomorrow and wanted to finish.

**Assessment & Plan:**

Ms. A is a 64-year-old man with a history of pulmonary embolism, type 2 diabetes, and exam findings of crackles, edema, and increased work of breathing who is admitted for further work-up of his fatigue and weakness in the setting of a recent pulmonary embolism.

**# Fatigue and Weakness:** The recent history of pulmonary embolism and exam findings of crackles, edema, and increased work of breathing make the fatigue and weakness most likely due to the pulmonary embolism. Several possible etiologies might explain the recurrent PEs including a very sedentary lifestyle, malignancy (such as pancreatic cancer but he has no abdominal discomfort), and age. Other possible causes for Mr. A’s fatigue and weakness include heart failure and lung cancer.

- Ultrasound of the heart.
- Surgery consultation for resecting pulmonary artery clots.
- Discussion with Mr. A to determine a longer-term plan for anticoagulation medication regimen that he finds workable with his preferences.

**#Health maintenance:**

- Will schedule follow-up with PCP to follow up on preventive care including colonoscopy and upcoming pneumococcal vaccination.
