# Supplementary material for: A Patient-Centered Documentation Skills Curriculum for Preclerkship Medical Students in an Open Notes Era
Source: MedEdPORTAL. 2024 Mar 26;20:11392. doi: 10.15766/mep_2374-8265.11392 (PMC10963659; doi:10.15766/mep_2374-8265.11392)
Supplement: Supplementary file 1 — Checklist of Best Practices.docxRubric.docxFacilitator Guide.docxCourse Planner Implementation Guide.docxAsynchronous Module folderStudent Guide.docxWritten Documentation Guide.docxStudent Session Slides.pptxSample Note.docxModel Note.docxAttitudinal Survey Questions.docxKnowledge Questions.docx [file mep_2374-8265.11392-s001.zip › H. Student Session Slides.pptx]

## Slide 1
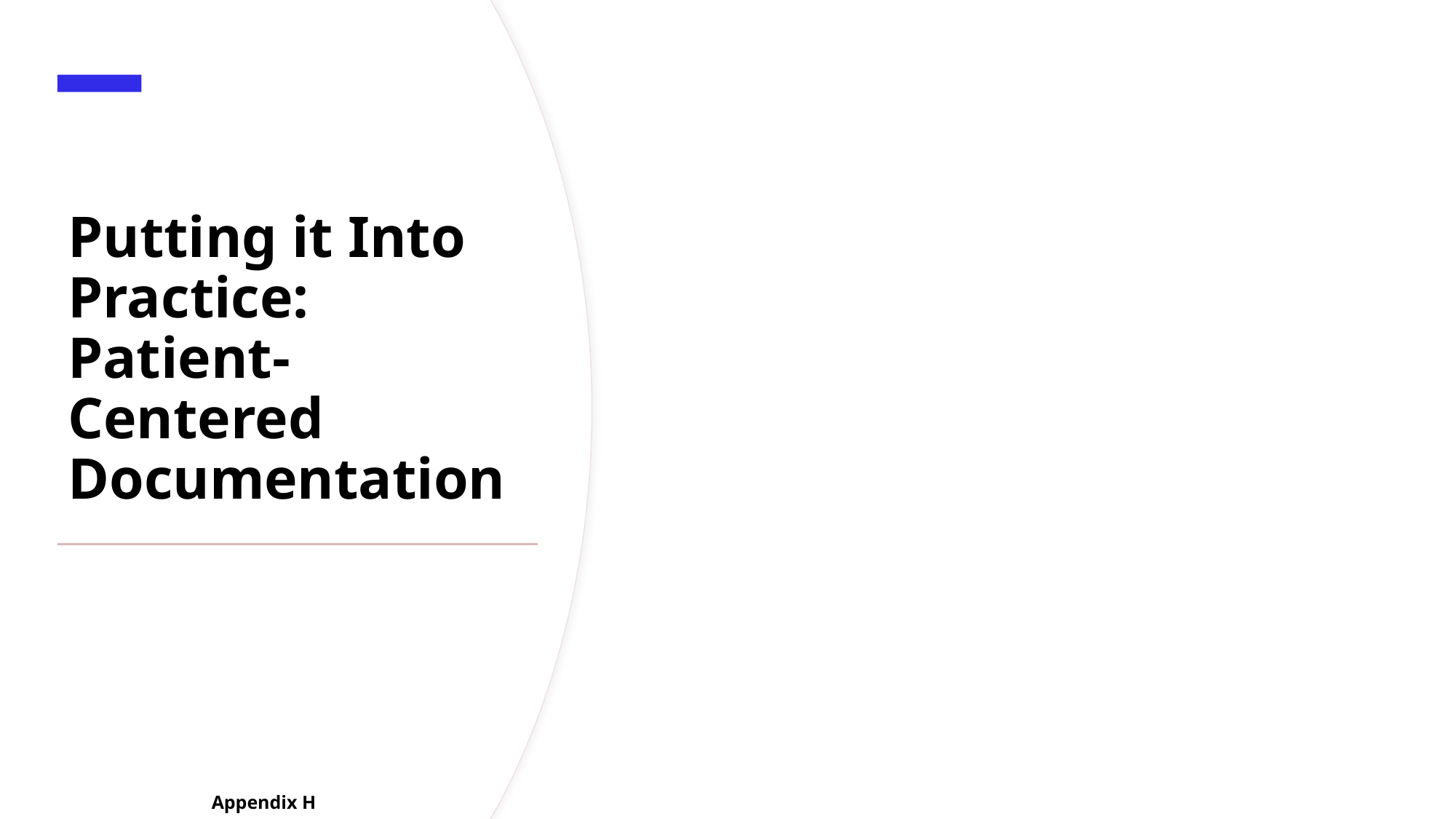

# Putting it Into Practice: Patient-Centered Documentation
Appendix H

## Slide 2
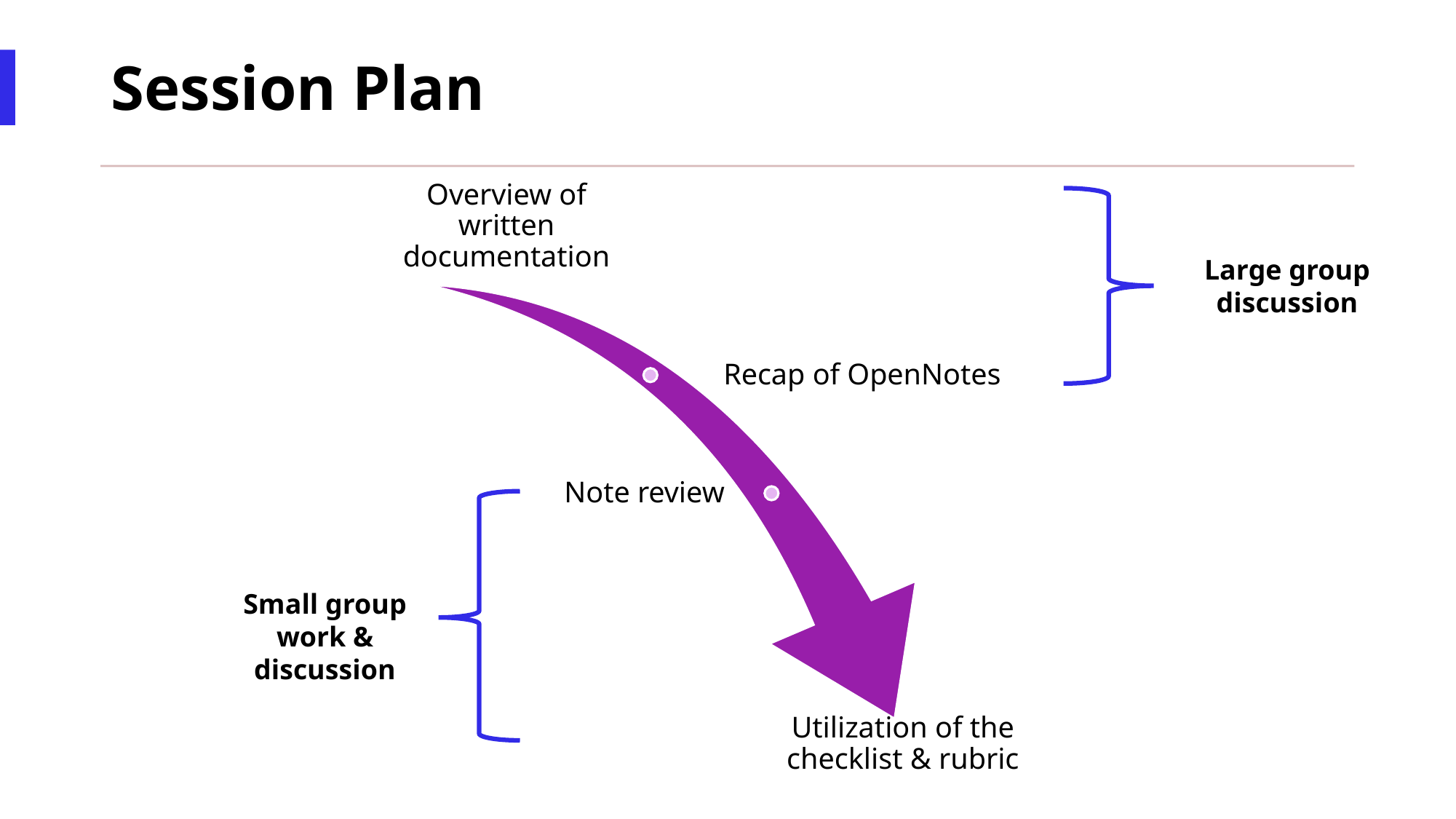

# Session Plan
Large group discussion
Small group work & discussion

## Slide 3
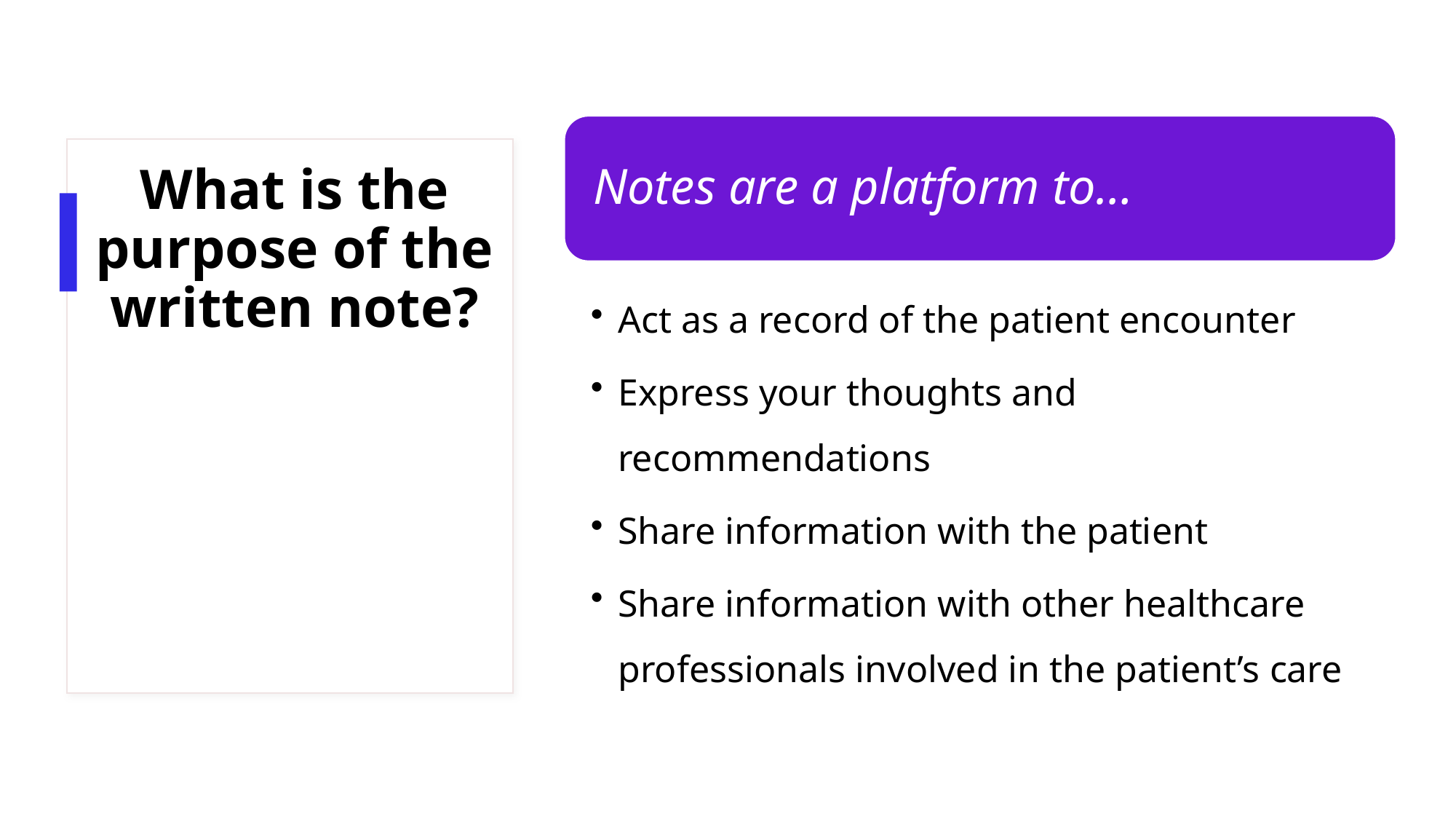

# What is the purpose of the written note?

## Slide 4
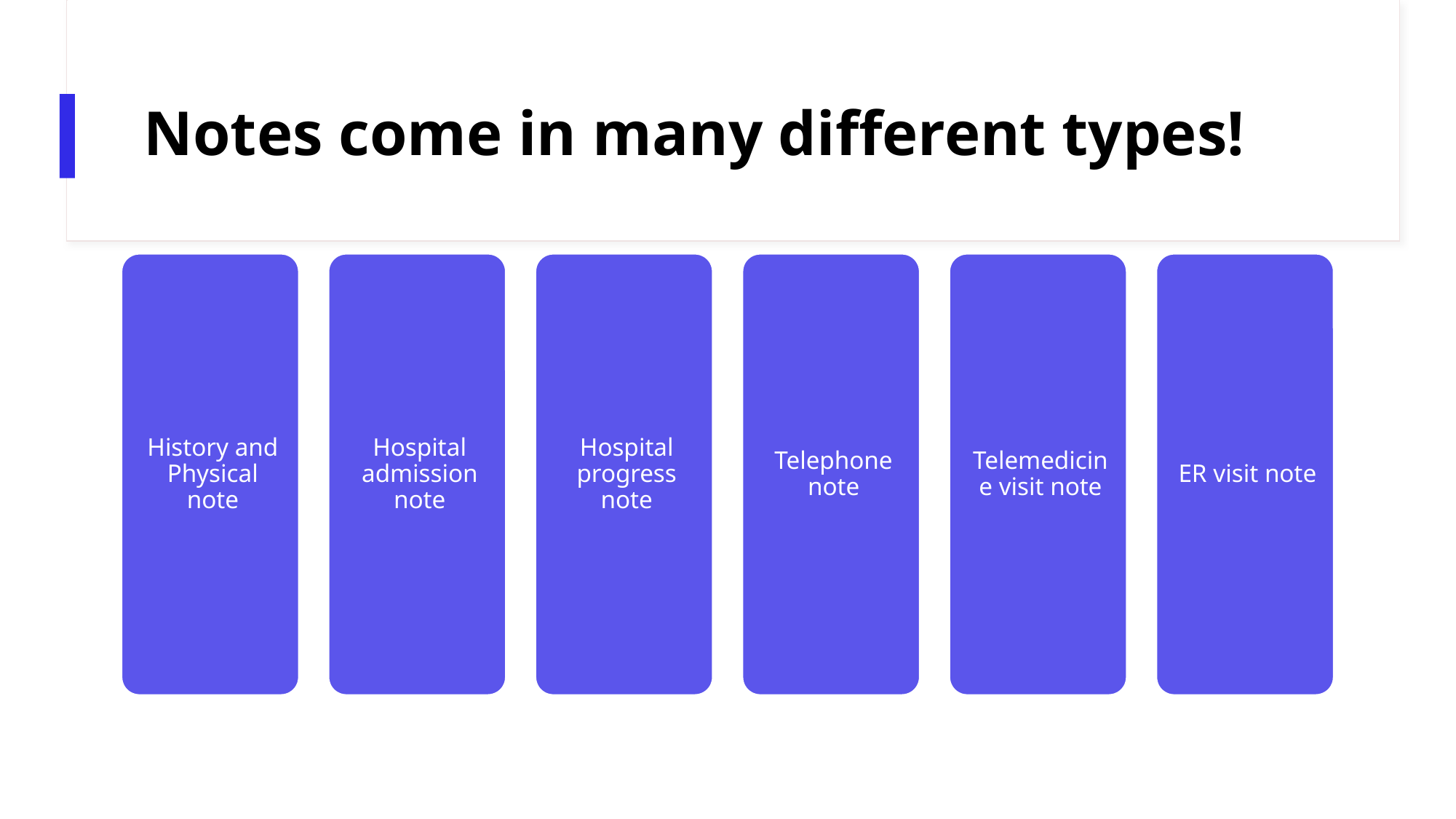

# Notes come in many different types!

## Slide 5
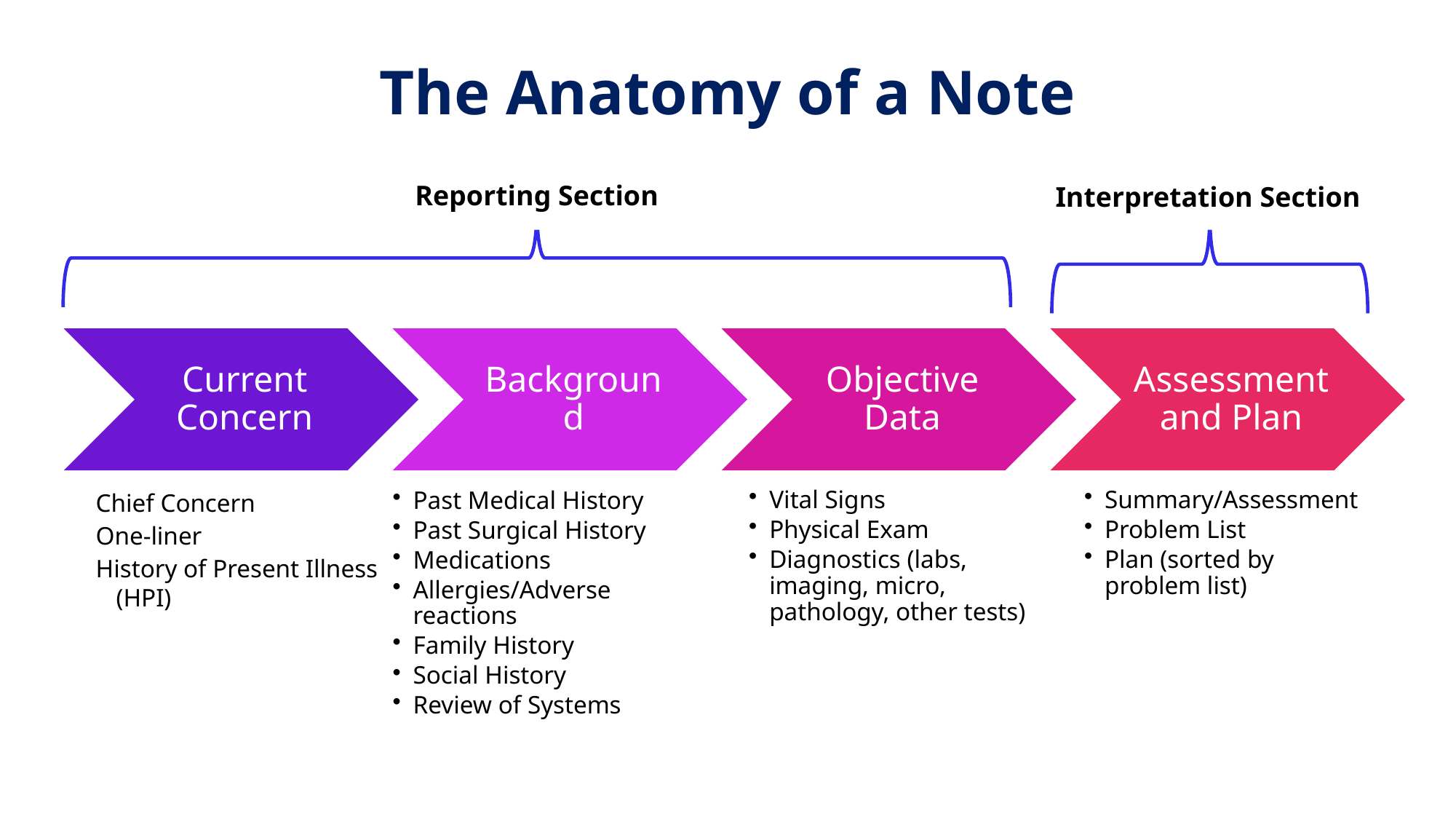

# The Anatomy of a Note
Reporting Section
Interpretation Section

## Slide 6
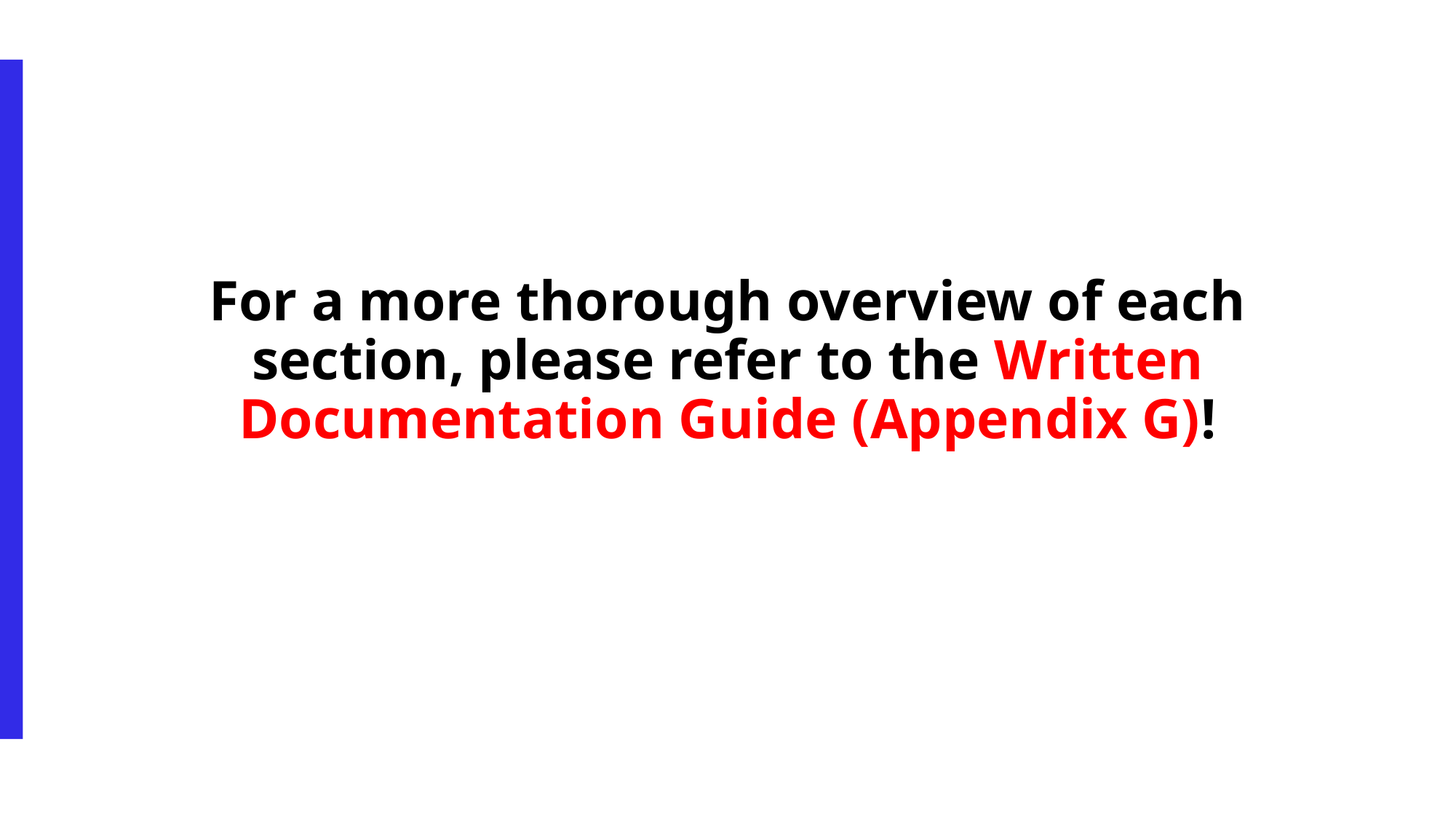

For a more thorough overview of each section, please refer to the Written Documentation Guide (Appendix G)!

## Slide 7
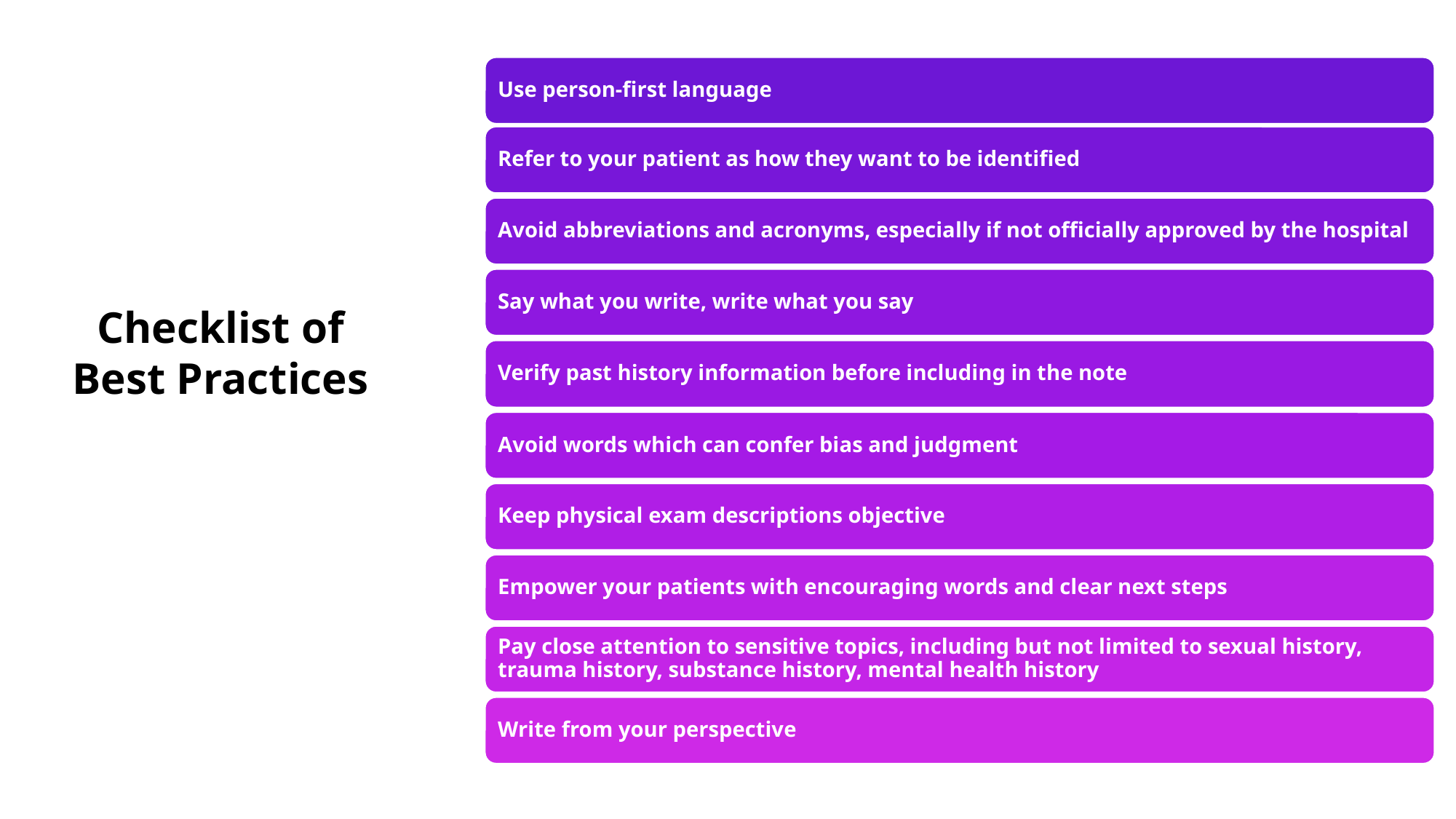

Checklist of Best Practices

## Slide 8
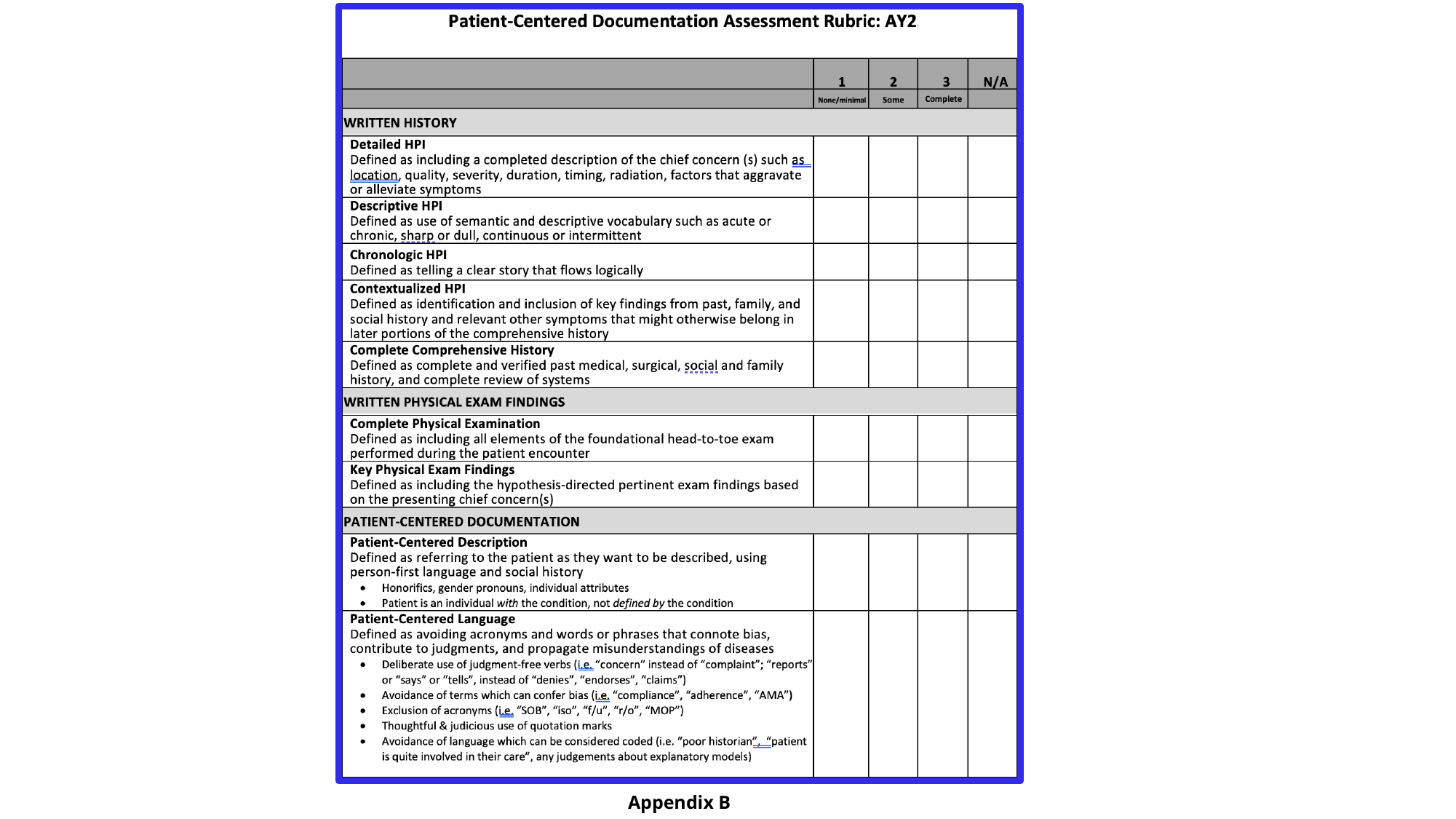

Appendix B

## Slide 9
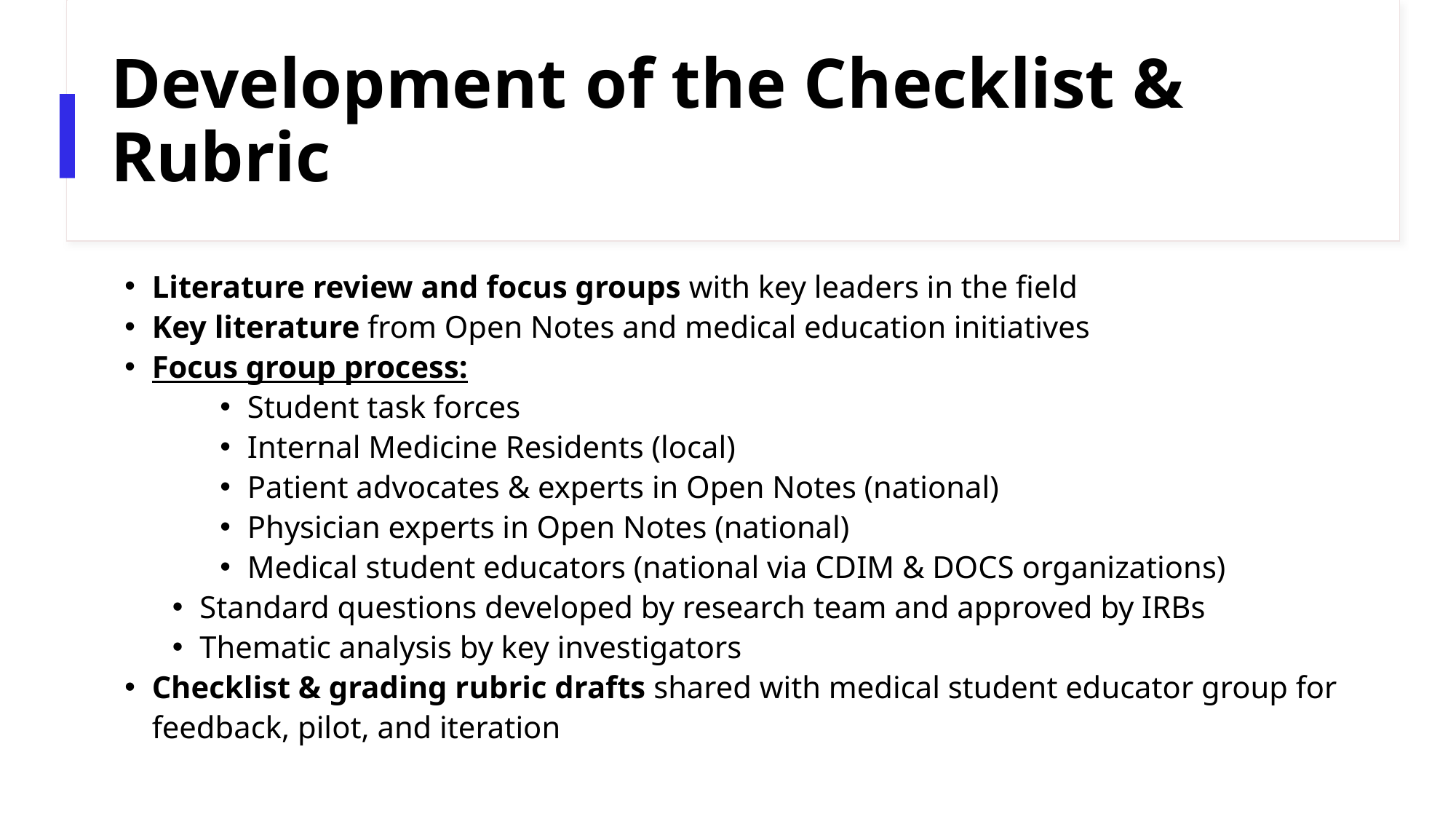

# Development of the Checklist & Rubric
Literature review and focus groups with key leaders in the field
Key literature from Open Notes and medical education initiatives
Focus group process:
Student task forces
Internal Medicine Residents (local)
Patient advocates & experts in Open Notes (national)
Physician experts in Open Notes (national)
Medical student educators (national via CDIM & DOCS organizations)
Standard questions developed by research team and approved by IRBs
Thematic analysis by key investigators
Checklist & grading rubric drafts shared with medical student educator group for feedback, pilot, and iteration

## Slide 10
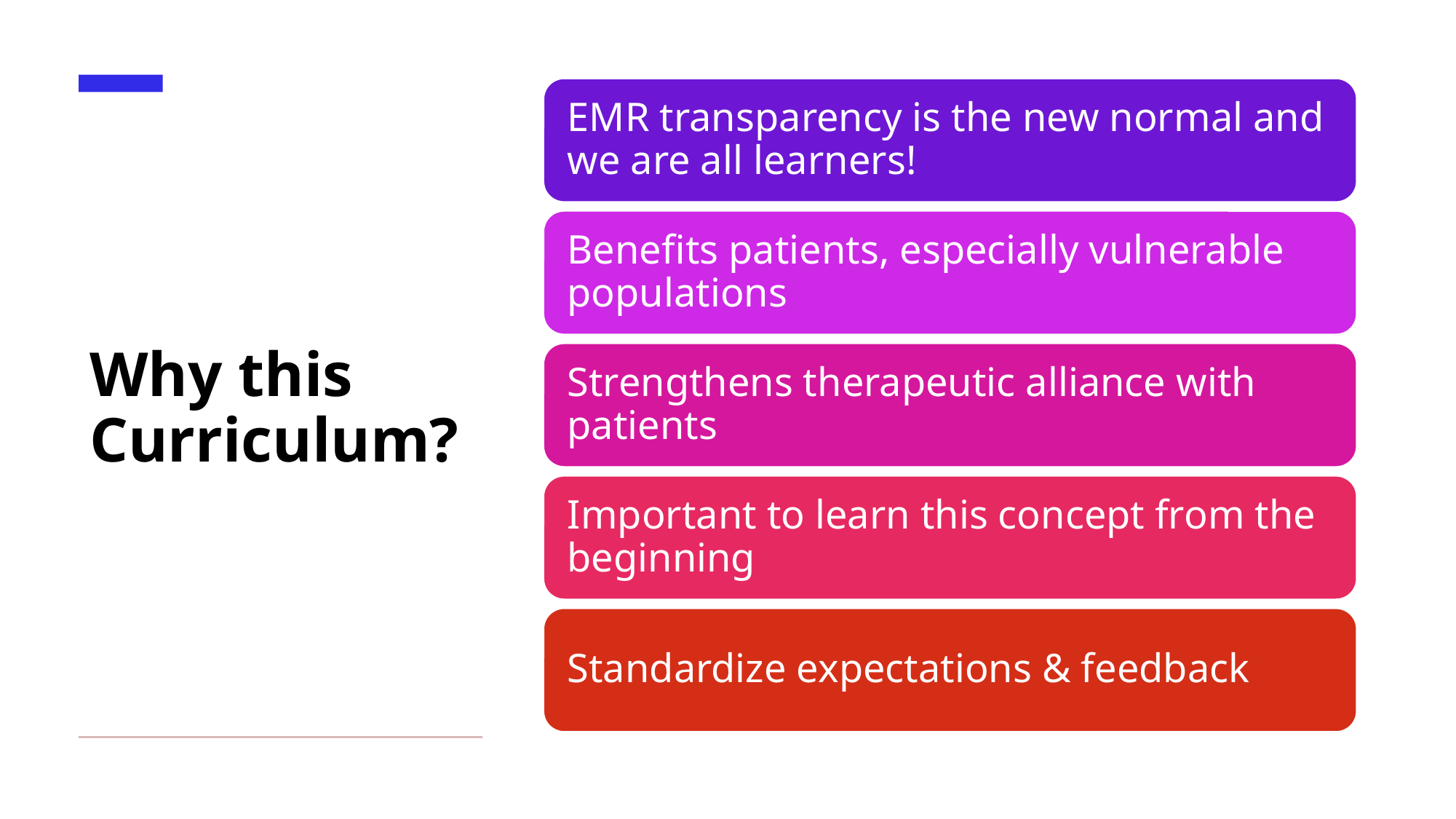

# Why this Curriculum?

## Slide 11
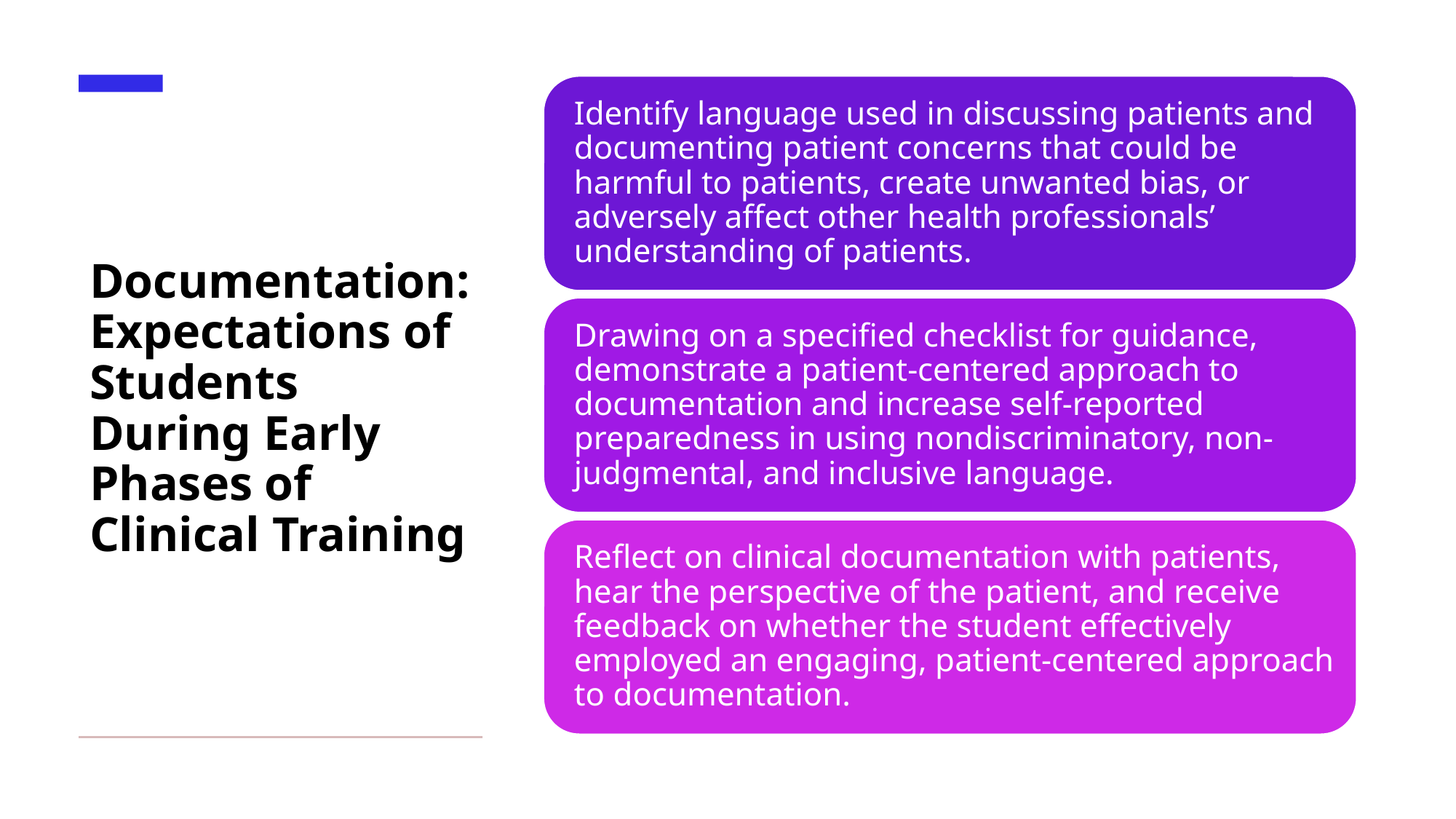

# Documentation: Expectations of Students During Early Phases of Clinical Training

## Slide 12
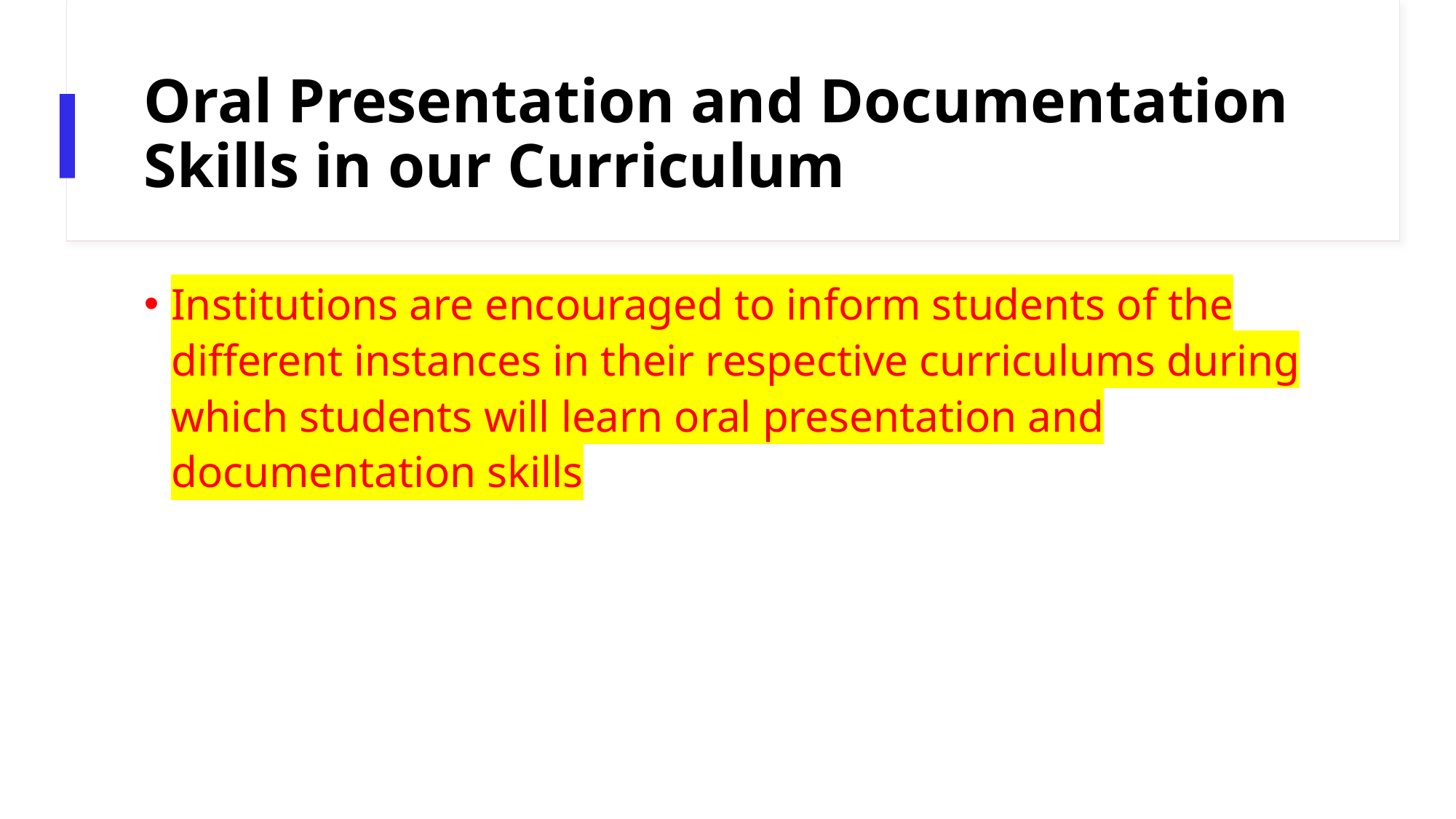

# Oral Presentation and Documentation Skills in our Curriculum
Institutions are encouraged to inform students of the different instances in their respective curriculums during which students will learn oral presentation and documentation skills

## Slide 13
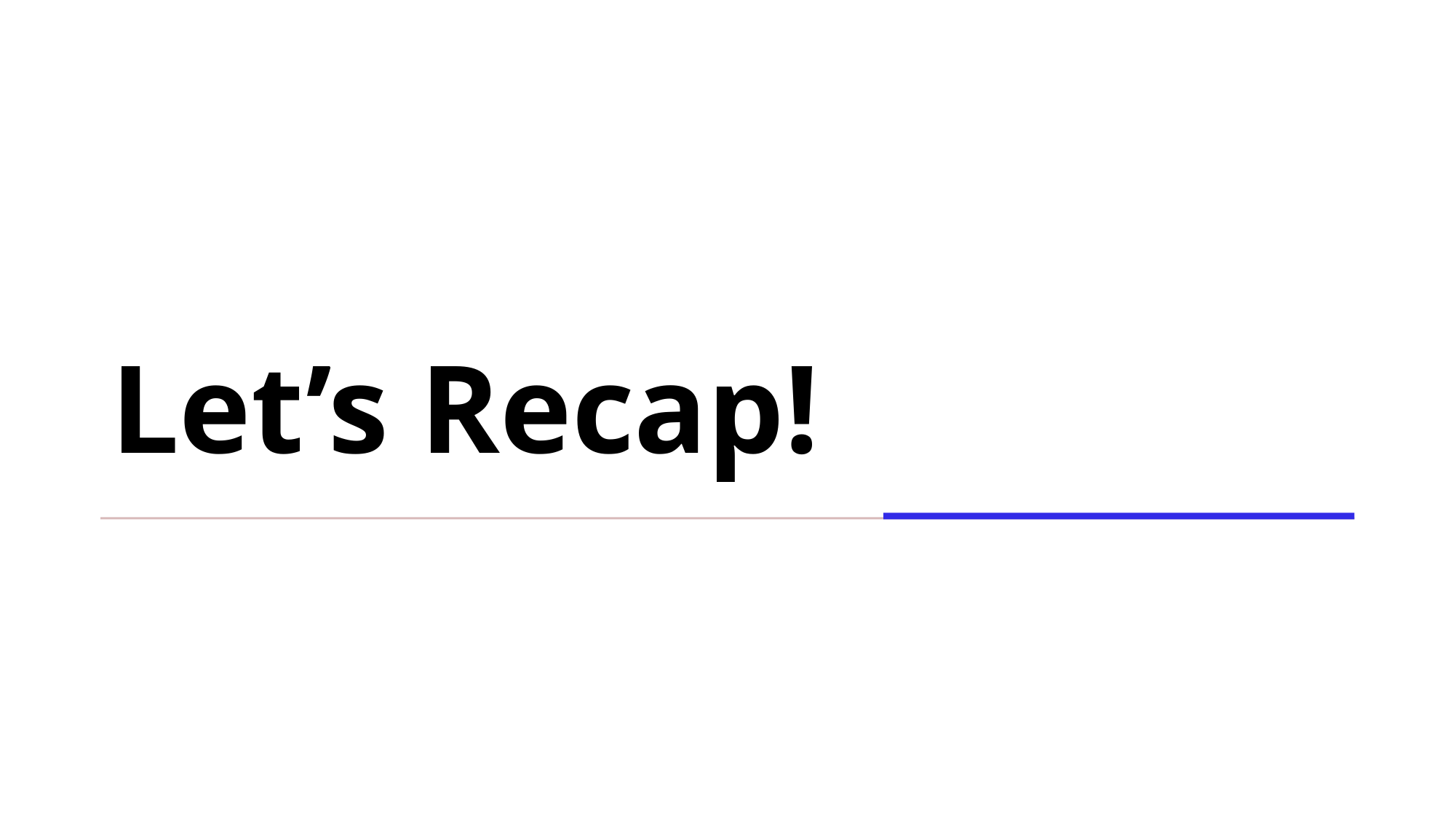

# Let’s Recap!

## Slide 14
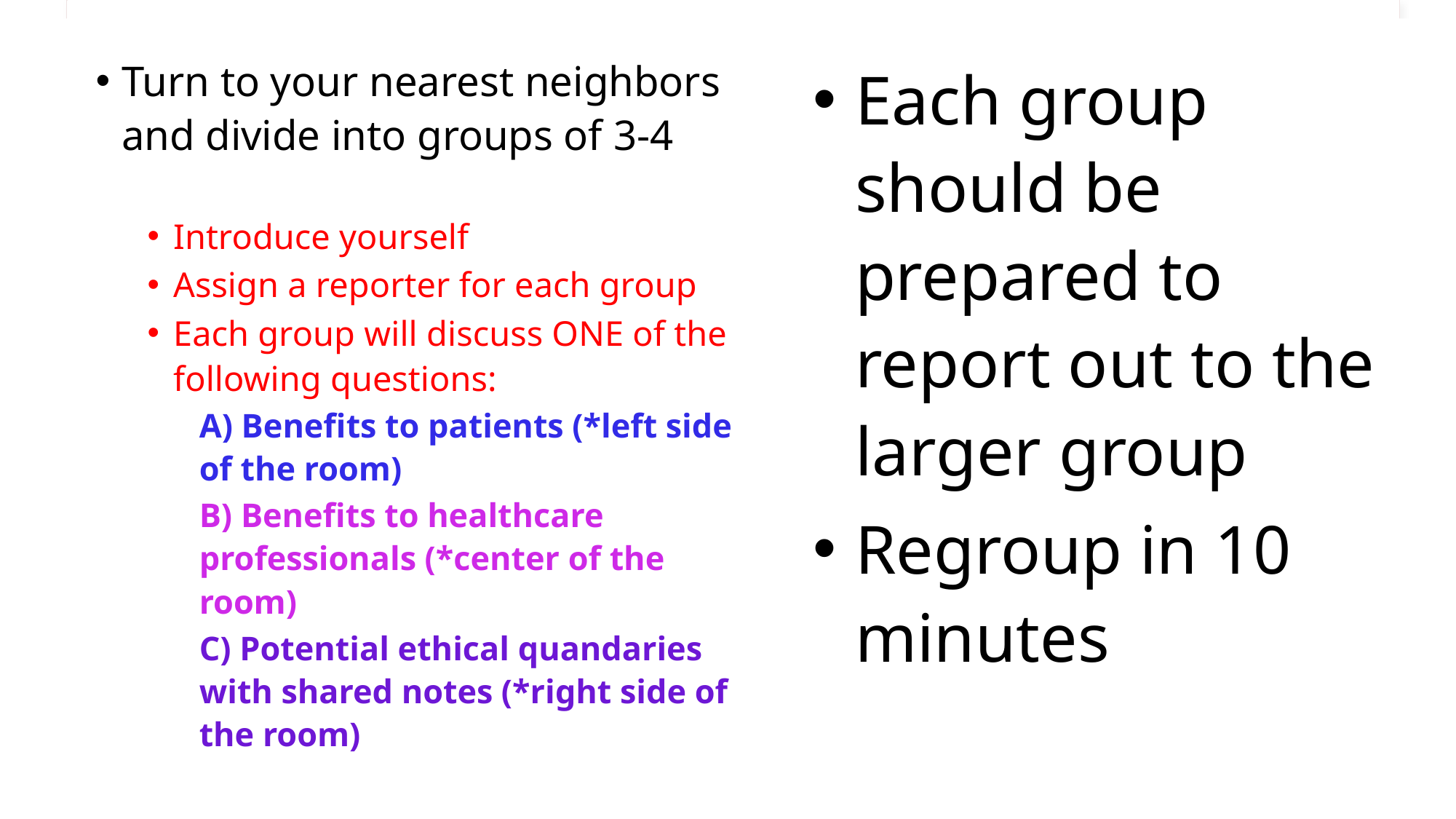

Turn to your nearest neighbors and divide into groups of 3-4
Introduce yourself
Assign a reporter for each group
Each group will discuss ONE of the following questions:
A) Benefits to patients (*left side of the room)
B) Benefits to healthcare professionals (*center of the room)
C) Potential ethical quandaries with shared notes (*right side of the room)
Each group should be prepared to report out to the larger group
Regroup in 10 minutes

## Slide 15
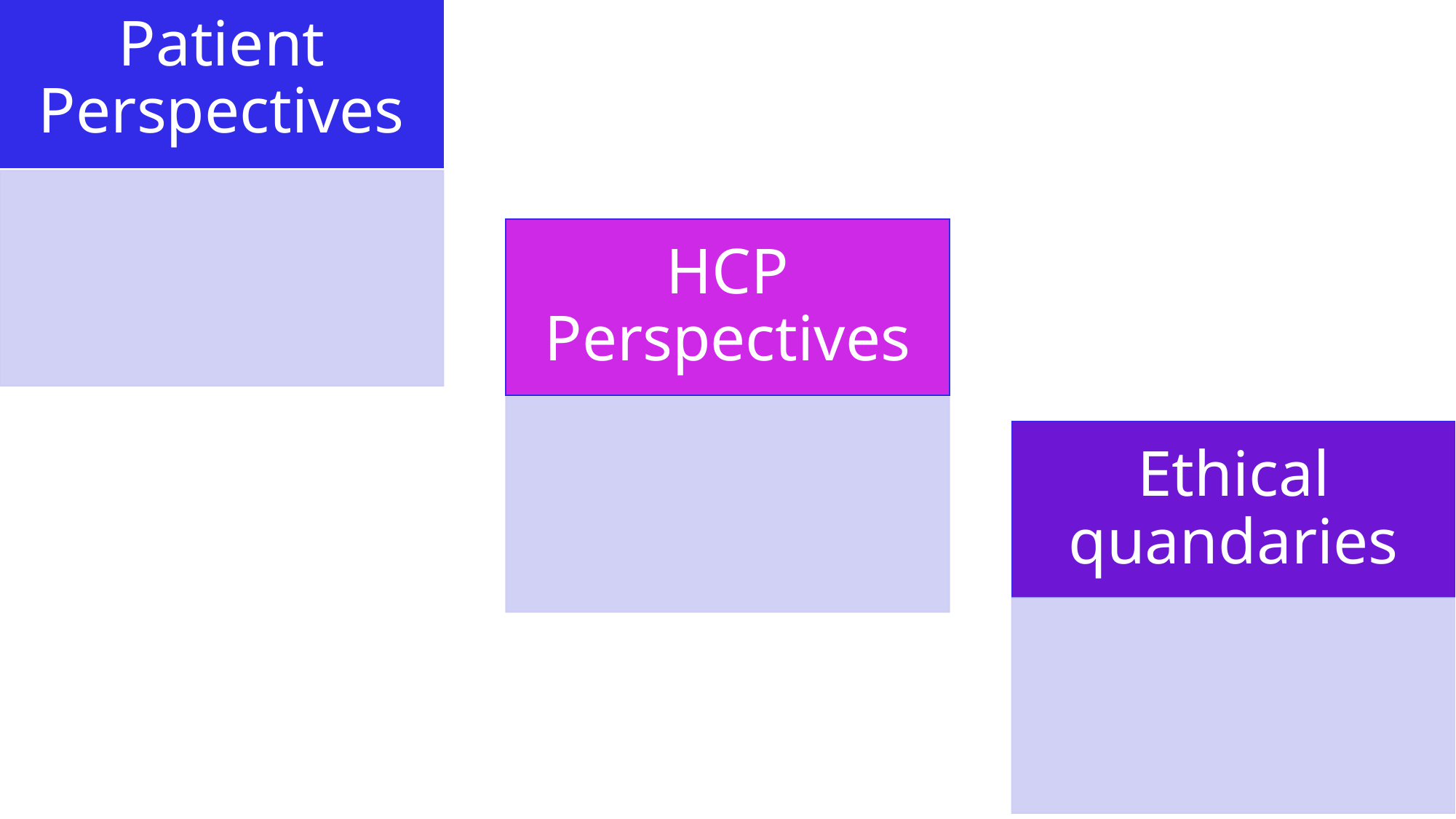

## Slide 16
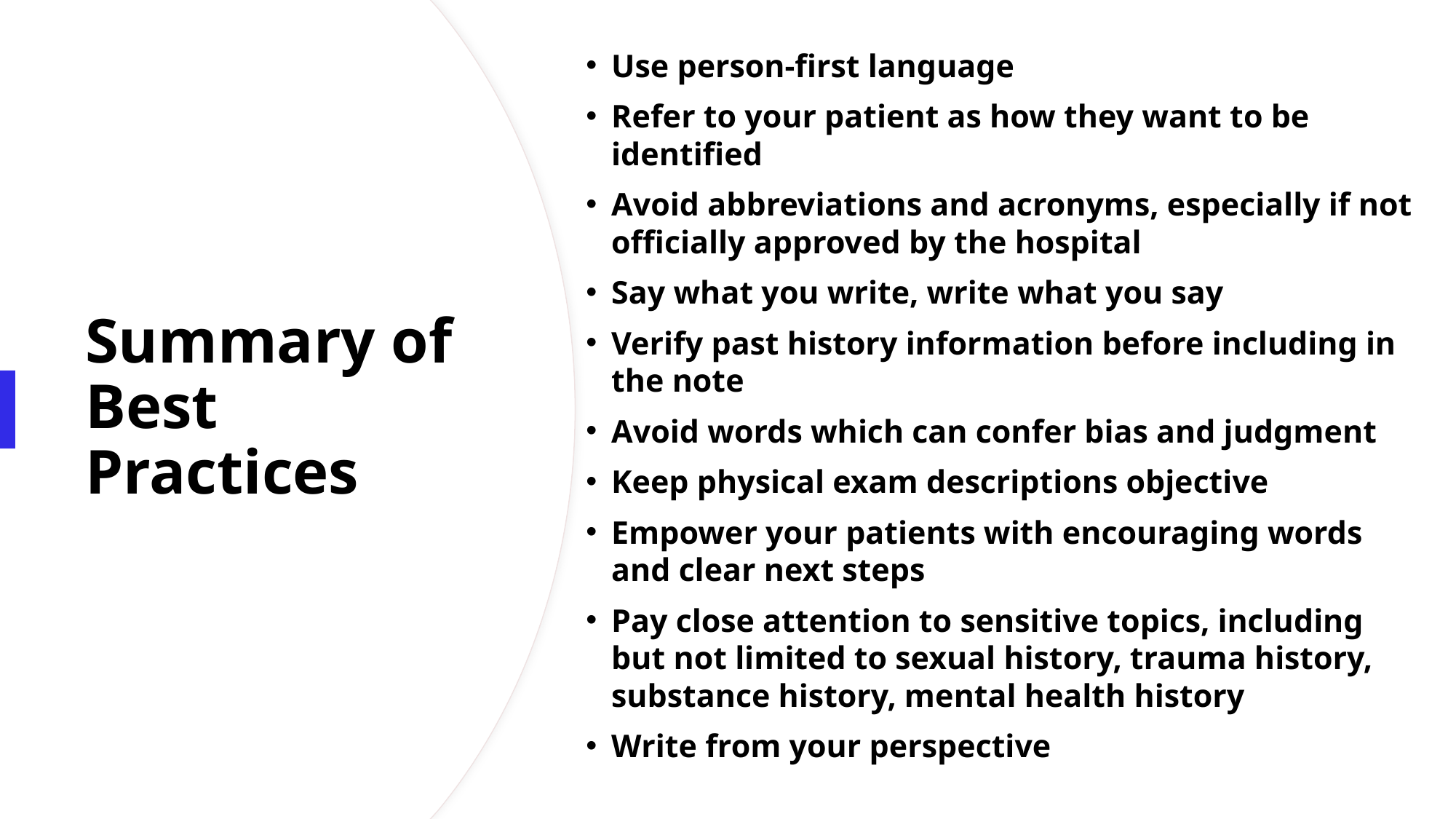

Use person-first language
Refer to your patient as how they want to be identified
Avoid abbreviations and acronyms, especially if not officially approved by the hospital
Say what you write, write what you say
Verify past history information before including in the note
Avoid words which can confer bias and judgment
Keep physical exam descriptions objective
Empower your patients with encouraging words and clear next steps
Pay close attention to sensitive topics, including but not limited to sexual history, trauma history, substance history, mental health history
Write from your perspective
# Summary of Best Practices

## Slide 17
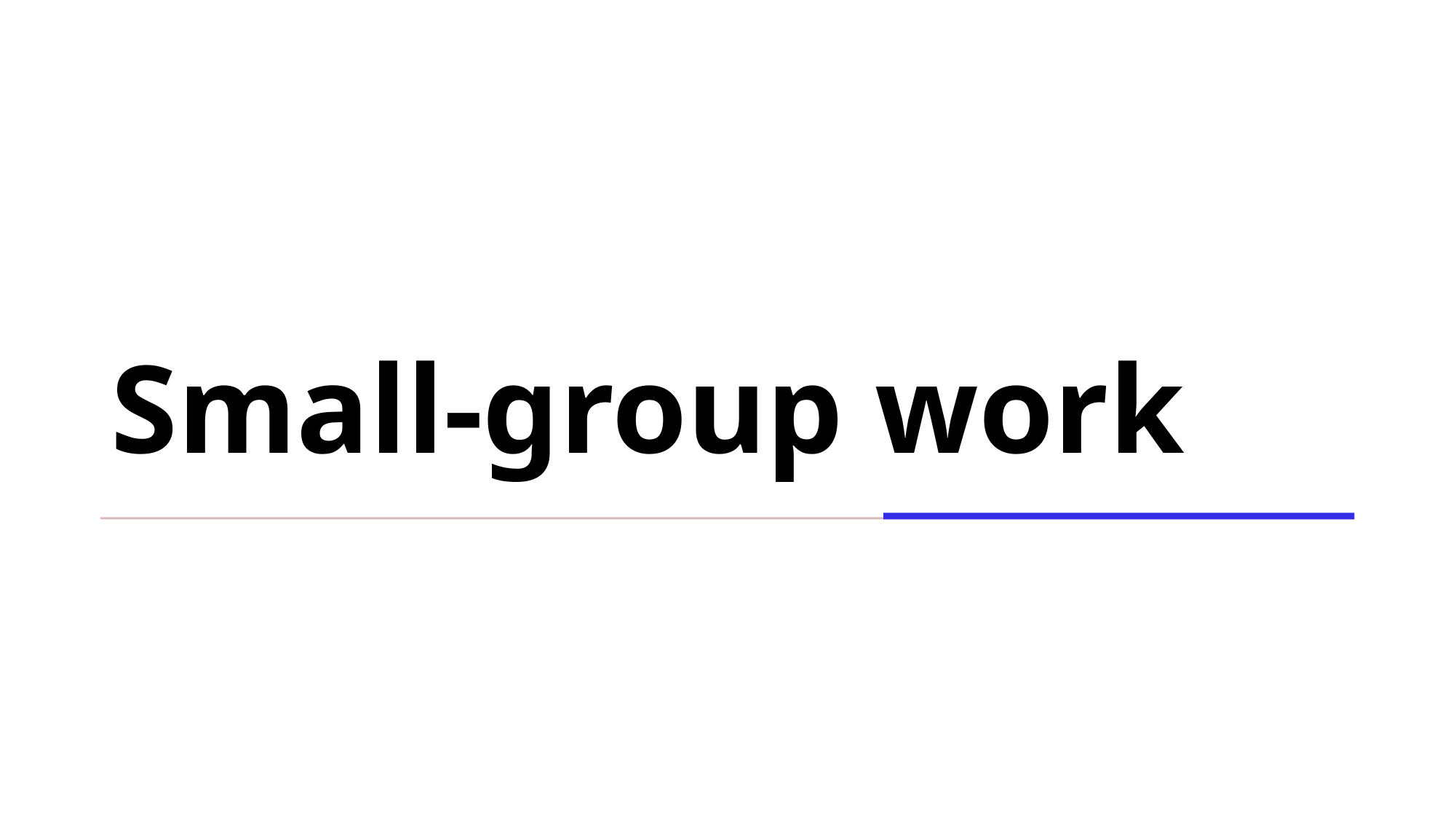

# Small-group work

## Slide 18
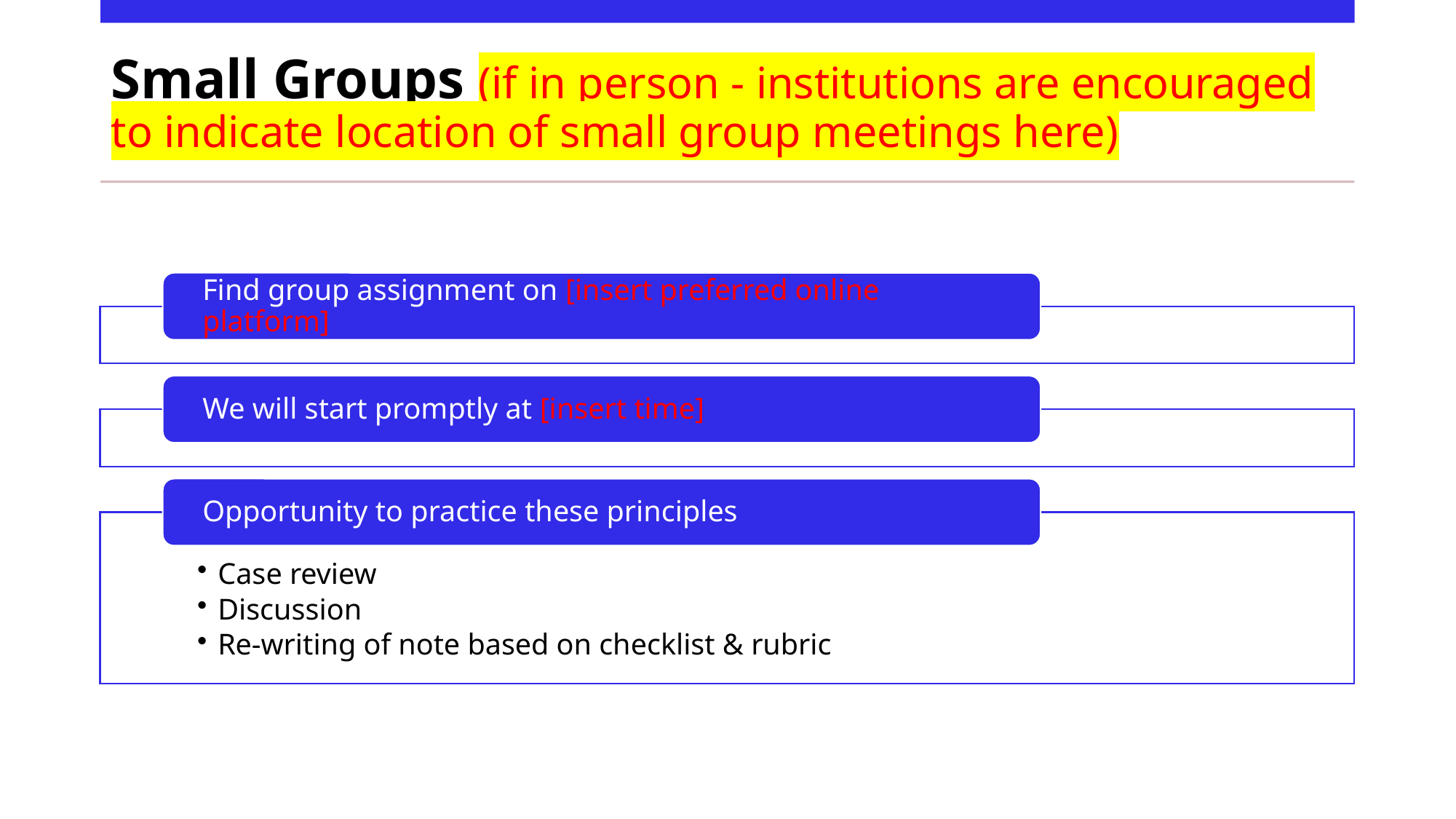

# Small Groups (if in person - institutions are encouraged to indicate location of small group meetings here)

## Slide 19
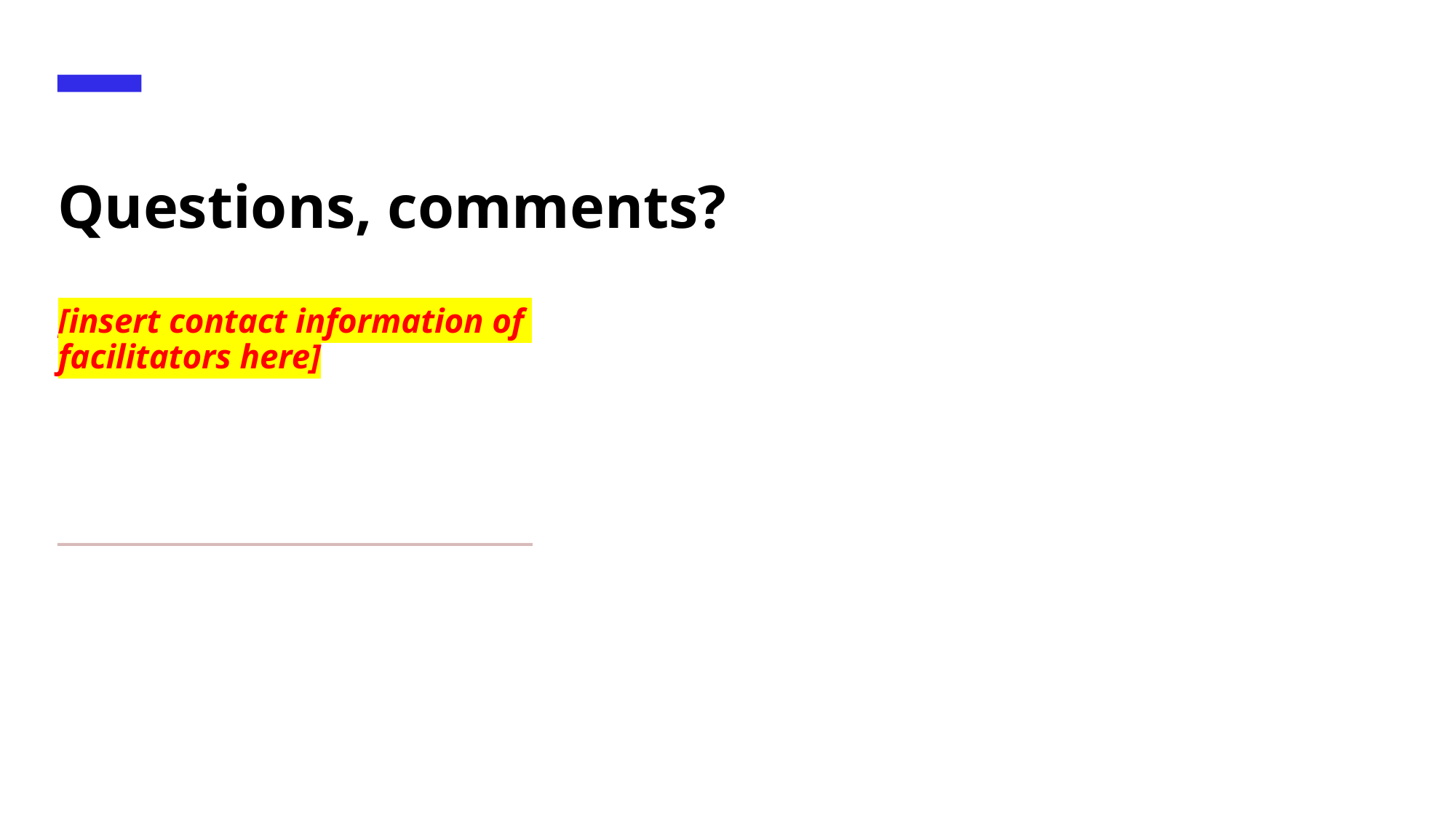

# Questions, comments? [insert contact information of facilitators here]
