# Supplementary material for: A Patient-Centered Documentation Skills Curriculum for Preclerkship Medical Students in an Open Notes Era
Source: MedEdPORTAL. 2024 Mar 26;20:11392. doi: 10.15766/mep_2374-8265.11392 (PMC10963659; doi:10.15766/mep_2374-8265.11392)
Supplement: Supplementary file 1 — Checklist of Best Practices.docxRubric.docxFacilitator Guide.docxCourse Planner Implementation Guide.docxAsynchronous Module folderStudent Guide.docxWritten Documentation Guide.docxStudent Session Slides.pptxSample Note.docxModel Note.docxAttitudinal Survey Questions.docxKnowledge Questions.docx [file mep_2374-8265.11392-s001.zip › I. Sample Note.docx]

*Appendix I: Sample Note*

*Time for activity: 60 minutes*

*For use during synchronous small group session*

**Sample Note**

Chief Complaint: Back pain

History of Present Illness:

Mr. S is a 52 y.o. IV drug user with a PMH of L sided PE and prior Hep C who presents with 2 days of back pain.

Insists he was briefly clean a few months ago but has since relapsed and acknowledges regular opiate use (fentanyl 2 to 3 grams daily). Last injected fentanyl into his neck this morning. He is prescribed suboxone for opioid substitution replacement therapy but only sporadically takes it, using it more to prevent withdrawal when he cannot shoot fentanyl/heroin.

Patient reports that he experienced bad pain in his back which started suddenly. The pain radiates down his leg. He initially presented to the MGH ED two days ago for evaluation but resisted medical advice, leaving AMA before imaging could be performed, even though he was well aware of the risks. He returned today when the pain became more unbearable. Per EMS, he had a bystander at a park call 911 for 10/10 back pain. Claims he has been unable to walk since the onset of pain two days ago. He also complains of bilateral wrist weakness and decreased grip strength for the last day. "It feels like I just can’t use my hands".

He is an intravenous drug addict and injects fentanyl mostly into his hands. He uses clean needles that he gets at the needle exchange. He likes to lick his needles. He never shares needles. He’s had a drug habit since age 12, first using crack cocaine; his drug of choice is now opioids. He’s demonstrated poor control in the past, being hospitalized most recently last month after falling down subway stairs- at that time was unmotivated to make change and refused to see the Addiction Consult Team. Alleges “he was clean then” but dirty urine tox screen proved otherwise.

He has never had an abscess in the past or been treated for bloodstream infection. He denies having any urinary incontinence, bowel incontinence, saddle anesthesia, leg numbness or tingling, or leg weakness. Also, no fever but had some chills. Claims no recent trauma.
