# Supplementary material for: A Patient-Centered Documentation Skills Curriculum for Preclerkship Medical Students in an Open Notes Era
Source: MedEdPORTAL. 2024 Mar 26;20:11392. doi: 10.15766/mep_2374-8265.11392 (PMC10963659; doi:10.15766/mep_2374-8265.11392)
Supplement: Supplementary file 1 — Checklist of Best Practices.docxRubric.docxFacilitator Guide.docxCourse Planner Implementation Guide.docxAsynchronous Module folderStudent Guide.docxWritten Documentation Guide.docxStudent Session Slides.pptxSample Note.docxModel Note.docxAttitudinal Survey Questions.docxKnowledge Questions.docx [file mep_2374-8265.11392-s001.zip › J. Model Note.docx]

*Appendix J: Model Note*

**Model Note**

Chief Concern: Back pain

History of Present Illness:

Mr. S is a 52 y.o. father, who identifies as male, and has a medical history notable for opioid use disorder (who currently engages in intravenous self-administration of fentanyl), as well as left-sided pulmonary embolism, and prior hepatitis C. He presents with 2 days of right-sided lower back pain.

Patient was in his usual state of health until two days ago when he reports suddenly experiencing an intense, 8/10 exploding pain in his right lower back. The pain radiates down his right leg and is present at all times. He finds minimal relief from flexing his hips. This pain has been accompanied by the inability to walk, bilateral wrist weakness, and decreased grip strength: "It feels like I just can’t use my hands".

He initially presented to the MGH ED two days ago for evaluation but left before imaging could be performed due to fear of missing out on his son’s college graduation, which took place later that day. He returned today when the pain became more unbearable. Per EMS, he had a bystander at a park call 911 for his back pain which escalated to a 10/10 on the pain scale.

Notably, he has a history of intravenous drug use for the past 30 years and currently injects fentanyl, mostly into his hands. He uses clean needles that he gets at the needle exchange. He shared he sometimes licks his needles, but never shares needles. Notably, patient’s onset of substance use was at age 12, stemming from a history of childhood neglect and trauma. He had used mostly crack cocaine when he was younger, but now uses opioids more often.

Patient shared that he was able to achieve abstinence from drugs a few months ago with the support of his recovery network in JP but has recently resumed regular opiate use (fentanyl 2 to 3 grams daily). He last injected fentanyl into his neck this morning. He is prescribed suboxone for medication-assisted treatment but takes it infrequently, using it more often as a means to prevent withdrawal when unable to use fentanyl/heroin. He was hospitalized most recently after falling down subway stairs. At that time, he had a positive urine tox screen but opted not to see the Addiction Consult Team.

He has never had an abscess in the past or been treated for bloodstream infection. He has not had any urinary incontinence, bowel incontinence, saddle anesthesia, leg numbness or tingling, or leg weakness. Also, he has not had fever, but has had some chills for the last day. No recent trauma.

Key Takeaways:

- Use person-first language
- Be cautious with abbreviations
- Tell a clear story in chronologic order that flows logically
- Avoid terms that suggest disbelief – consider replacing terms like “insists”, “denies”, or “alleges” for terms such as “reports,” “says,” or “tells.”
- Refrain from using terms that can confer bias and perpetuate negative stereotypes
- Use more detail and descriptors to characterize the chief concern
