# Supplementary material for: A Patient-Centered Documentation Skills Curriculum for Preclerkship Medical Students in an Open Notes Era
Source: MedEdPORTAL. 2024 Mar 26;20:11392. doi: 10.15766/mep_2374-8265.11392 (PMC10963659; doi:10.15766/mep_2374-8265.11392)
Supplement: Supplementary file 1 — Checklist of Best Practices.docxRubric.docxFacilitator Guide.docxCourse Planner Implementation Guide.docxAsynchronous Module folderStudent Guide.docxWritten Documentation Guide.docxStudent Session Slides.pptxSample Note.docxModel Note.docxAttitudinal Survey Questions.docxKnowledge Questions.docx [file mep_2374-8265.11392-s001.zip › K. Attitudinal Survey Questions.docx]

*Appendix K: Attitudinal Survey Questions*

1. In your opinion, how important is writing a patient-centered note for effective patient care?
   1. Very prepared
   2. Somewhat prepared
   3. Neutral
   4. Somewhat unprepared
   5. Very unprepared
2. How prepared do you feel to write a clinical note (without consideration of patient readers)?
   1. Very prepared
   2. Somewhat prepared
   3. Neutral
   4. Somewhat unprepared
   5. Very unprepared
3. How prepared do you feel to write a patient-centered note?
   1. Very prepared
   2. Somewhat prepared
   3. Neutral
   4. Somewhat unprepared
   5. Very unprepared
4. How prepared do you feel to talk to patients (and/or families) about their clinical note?
   1. Very prepared
   2. Somewhat prepared
   3. Neutral
   4. Somewhat unprepared
   5. Very unprepared
5. Please rate the effectiveness of the session on Patient-Centered Documentation.
   1. Excellent
   2. Good
   3. Average
   4. Fair
   5. Poor
6. Please provide specific comments on the Patient-Centered Documentation session:
   1. Strengths of the session:
   2. Opportunities for improving the session:
