# Supplementary material for: A Patient-Centered Documentation Skills Curriculum for Preclerkship Medical Students in an Open Notes Era
Source: MedEdPORTAL. 2024 Mar 26;20:11392. doi: 10.15766/mep_2374-8265.11392 (PMC10963659; doi:10.15766/mep_2374-8265.11392)
Supplement: Supplementary file 1 — Checklist of Best Practices.docxRubric.docxFacilitator Guide.docxCourse Planner Implementation Guide.docxAsynchronous Module folderStudent Guide.docxWritten Documentation Guide.docxStudent Session Slides.pptxSample Note.docxModel Note.docxAttitudinal Survey Questions.docxKnowledge Questions.docx [file mep_2374-8265.11392-s001.zip › L. Knowledge Questions.docx]

*Appendix L: Knowledge Questions*

1. Select the one-line phrase that best illustrates person-first language:
   1. Mr. F is a 43-year-old with IVDU presenting with new foot pain for one week.
   2. Mr. F is a 43-year-old diabetic (last a1c 8.2%) here for follow-up on his diabetes.
   3. **Mr. F is a 43-year-old engineer experiencing a recurrence of substance use disorder for the last 1 month and presents to discuss this today.**
   4. Mr. F is a 43-year-old diabetic with non-compliance to insulin, who presents with hyperglycemia for 1 day.
2. Which of the following phrases in a clinical note is least likely to convey bias?
   1. **She tells me she has had difficulty with the amount of alcohol she has recently been consuming daily.**
   2. She denies use of alcohol, tobacco products, or recreational drugs.
   3. She admits to drinking 2 glasses of wine daily.
   4. She has had 5 detox attempts with relapses this past year.
3. Which of the following is true?
   1. Findings from the OpenNotes pilot study in 2010 showed that opening up clinical notes to patients led to decreased patient trust, empowerment, adherence, and health literacy.
   2. **The Interoperability & Information Blocking Rule in 2021 requires all healthcare providers to give patients access to all health information in their EMR without delay and without change.**
   3. In general, patients who read their notes report increased confusion understanding their medical condition and feeling in less control of their care.
   4. It has been shown to be harmful to patients with mental health conditions when accessing their notes.
