# Supplementary figures and images for: A Patient-Centered Documentation Skills Curriculum for Preclerkship Medical Students in an Open Notes Era
Source: MedEdPORTAL. 2024 Mar 26;20:11392. doi: 10.15766/mep_2374-8265.11392 (PMC10963659; doi:10.15766/mep_2374-8265.11392)

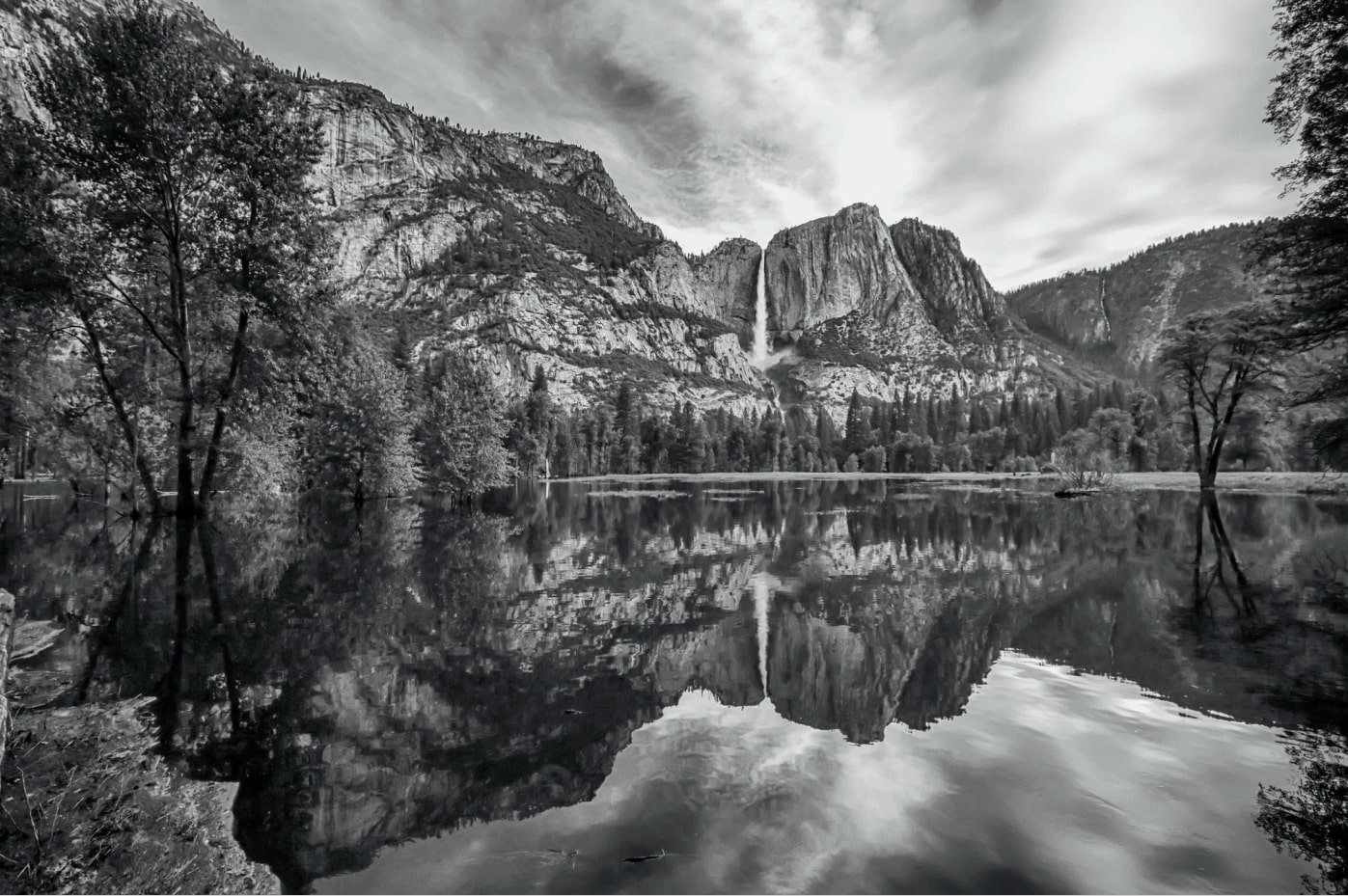

Supplement: Supplementary file 1 — Checklist of Best Practices.docxRubric.docxFacilitator Guide.docxCourse Planner Implementation Guide.docxAsynchronous Module folderStudent Guide.docxWritten Documentation Guide.docxStudent Session Slides.pptxSample Note.docxModel Note.docxAttitudinal Survey Questions.docxKnowledge Questions.docx [file mep_2374-8265.11392-s001.zip › E. Asynchronous Module/content/assets/3_bDAvGl1zMhNXdj_example-header-image.jpg]

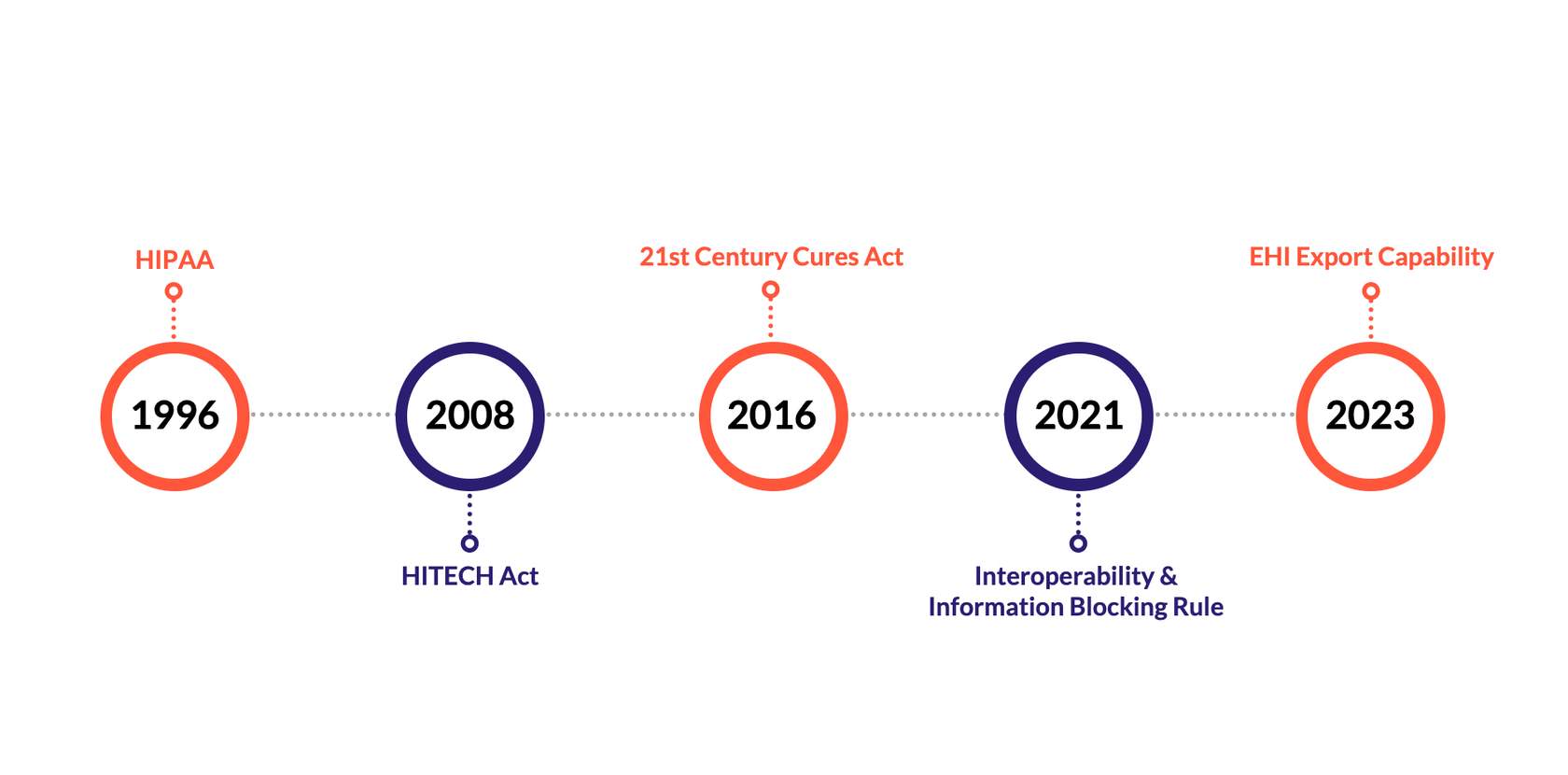

Supplement: Supplementary file 1 — Checklist of Best Practices.docxRubric.docxFacilitator Guide.docxCourse Planner Implementation Guide.docxAsynchronous Module folderStudent Guide.docxWritten Documentation Guide.docxStudent Session Slides.pptxSample Note.docxModel Note.docxAttitudinal Survey Questions.docxKnowledge Questions.docx [file mep_2374-8265.11392-s001.zip › E. Asynchronous Module/content/assets/A-YsvM7RTLjSfVPF_T_kLyq8b4jvb7s55.jpg]

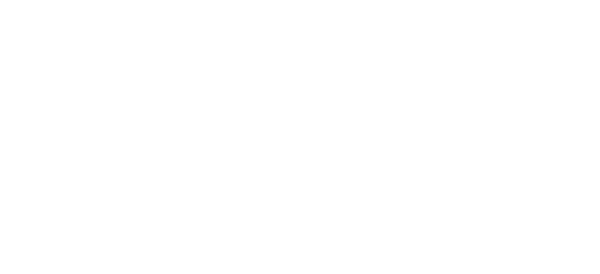

Supplement: Supplementary file 1 — Checklist of Best Practices.docxRubric.docxFacilitator Guide.docxCourse Planner Implementation Guide.docxAsynchronous Module folderStudent Guide.docxWritten Documentation Guide.docxStudent Session Slides.pptxSample Note.docxModel Note.docxAttitudinal Survey Questions.docxKnowledge Questions.docx [file mep_2374-8265.11392-s001.zip › E. Asynchronous Module/content/assets/FAxloEJ6kewozGCv_Y37VhTyhK4lmGmhc.gif]

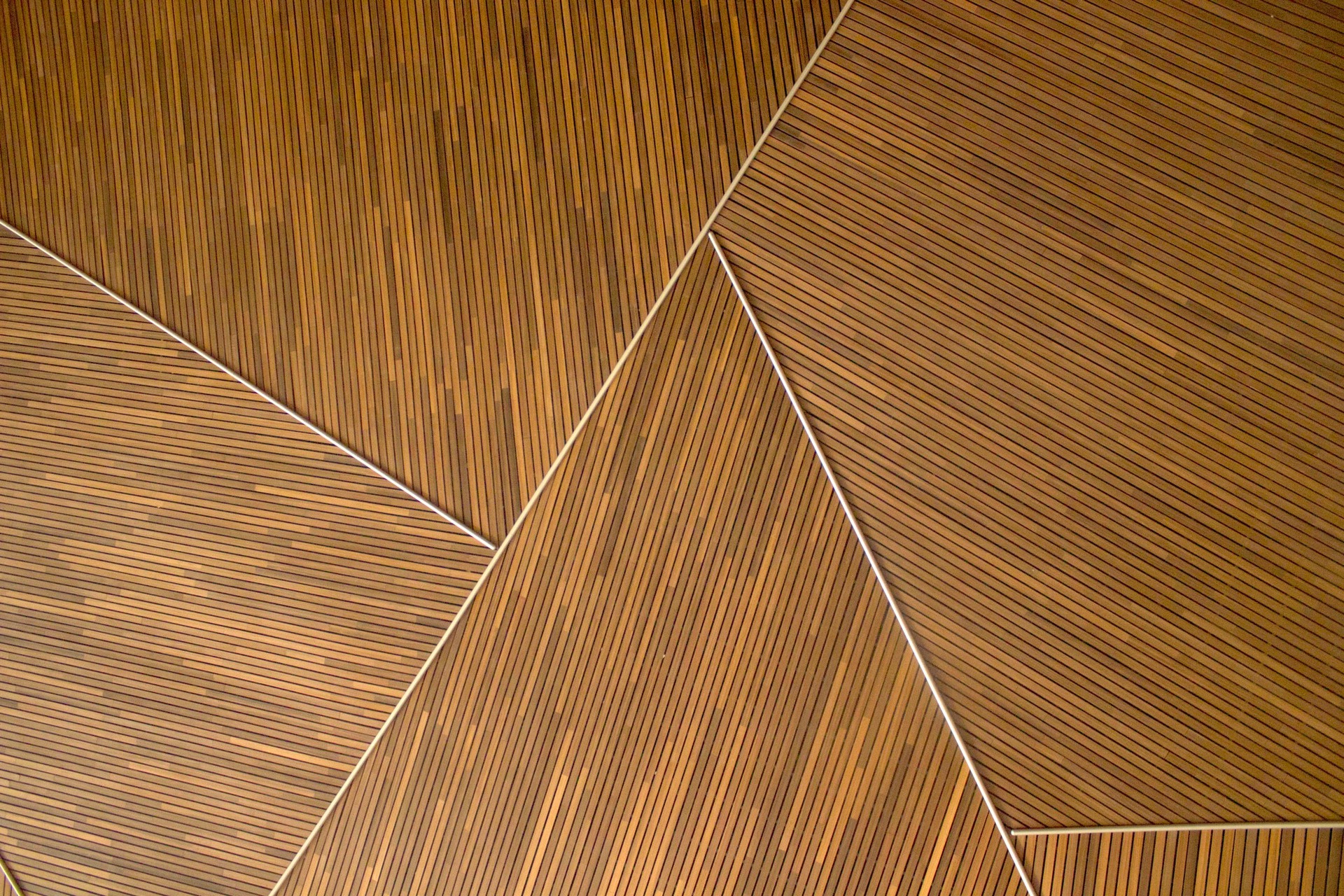

Supplement: Supplementary file 1 — Checklist of Best Practices.docxRubric.docxFacilitator Guide.docxCourse Planner Implementation Guide.docxAsynchronous Module folderStudent Guide.docxWritten Documentation Guide.docxStudent Session Slides.pptxSample Note.docxModel Note.docxAttitudinal Survey Questions.docxKnowledge Questions.docx [file mep_2374-8265.11392-s001.zip › E. Asynchronous Module/content/assets/hs6PSo7CqwcxKBQg_35_abstract.jpg]

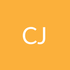

Supplement: Supplementary file 1 — Checklist of Best Practices.docxRubric.docxFacilitator Guide.docxCourse Planner Implementation Guide.docxAsynchronous Module folderStudent Guide.docxWritten Documentation Guide.docxStudent Session Slides.pptxSample Note.docxModel Note.docxAttitudinal Survey Questions.docxKnowledge Questions.docx [file mep_2374-8265.11392-s001.zip › E. Asynchronous Module/content/assets/Y2AToKO871Zx6MDX_small.png]

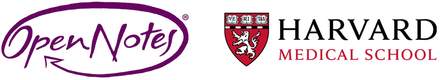

Supplement: Supplementary file 1 — Checklist of Best Practices.docxRubric.docxFacilitator Guide.docxCourse Planner Implementation Guide.docxAsynchronous Module folderStudent Guide.docxWritten Documentation Guide.docxStudent Session Slides.pptxSample Note.docxModel Note.docxAttitudinal Survey Questions.docxKnowledge Questions.docx [file mep_2374-8265.11392-s001.zip › E. Asynchronous Module/content/assets/ze9uPRjAn6UjoqRR_839N98LLJLIZL_JJ.jpg]
